# Surfactant-guided spatial assembly of nano-architectures for molecular profiling of extracellular vesicles

**DOI:** 10.1038/s41467-021-23759-9

**Published:** 2021-06-30

**Authors:** Zhigang Wang, Haitao Zhao, Yan Zhang, Auginia Natalia, Chin-Ann J. Ong, Melissa C. C. Teo, Jimmy B. Y. So, Huilin Shao

**Affiliations:** 1 Institute for Health Innovation & Technology, National University of Singapore, Singapore, Singapore; 2 Department of Biomedical Engineering, Faculty of Engineering, National University of Singapore, Singapore, Singapore; 3 Division of Surgical Oncology, National Cancer Centre, Singapore, Singapore; 4 Department of Surgery, Yong Loo Lin School of Medicine, National University of Singapore, Singapore, Singapore; 5 Division of Surgical Oncology, National University Cancer Institute, Singapore, Singapore; 6 Institute of Molecular and Cell Biology, Agency for Science, Technology and Research, Singapore, Singapore

**Keywords:** Biosensors, Nanobiotechnology, Metal-organic frameworks, Nanoscale materials

## Abstract

The controlled assembly of nanomaterials into desired architectures presents many opportunities; however, current preparations lack spatial precision and versatility in developing complex nano-architectures. Inspired by the amphiphilic nature of surfactants, we develop a facile approach to guide nanomaterial integration – spatial organization and distribution – in metal-organic frameworks (MOFs). Named surfactant tunable spatial architecture (STAR), the technology leverages the varied interactions of surfactants with nanoparticles and MOF constituents, respectively, to direct nanoparticle arrangement while molding the growing framework. By surfactant matching, the approach achieves not only tunable and precise integration of diverse nanomaterials in different MOF structures, but also fast and aqueous synthesis, in solution and on solid substrates. Employing the approach, we develop a dual-probe STAR that comprises peripheral working probes and central reference probes to achieve differential responsiveness to biomarkers. When applied for the direct profiling of clinical ascites, STAR reveals glycosylation signatures of extracellular vesicles and differentiates cancer patient prognosis.

## Abstract (web summary)

Current methods for controlled assembly of nanomaterials into desired architectures often lack the precision and versatility to develop complex architectures. Here the authors report STAR, surfactant tunable spatial architecture, to guide nanomaterial integration in metal-organic frameworks.

## Introduction

The assembly of functional nanomaterials into desired architectures has attracted considerable attention. The properties of these architectures depend on not only the characteristics of individual nanoparticle building blocks but also the spatial geometry of the assemblies to achieve unprecedented performance that surpasses those of the constituents^1–3^. While nanoparticles of different material composition, shape, and size can be precisely engineered to achieve diverse functionalities^4–6^, their controlled assembly and spatial organization remain a challenging task to realize sophisticated architectures.

Several approaches have been developed to improve the integration control. For example, through colloidal self-assembly, nanoparticles modified with distinct, complementary molecules (e.g., proteins and nucleic acids) can organize themselves via various assembly forces (e.g., attractive and repulsive forces)^7–10^. Despite its high specificity, the approach requires dedicated, sequence-specific modifications and becomes increasingly challenging to multiplex. To improve the incorporation versatility, external templates are used to assemble nanoparticles into hybrid architectures^11,12^. In particular, metal–organic frameworks (MOFs) have crystalline structures, uniform cavities and tunable properties, making them an attractive matrix to host nanoparticles^13–16^. Nevertheless, existing preparation approaches lack spatial precision to control nanoparticle distribution and organization within the frameworks. Nanoparticles are synthesized in situ in pre-formed MOFs^17,18^ or MOFs are formed around pre-synthesized nanoparticles^19,20^; both approaches result in passive integration and random nanoparticle distribution. While new strategies have been developed to improve the spatial control, through in situ transformation of metal oxides^21^ or stepwise addition of nanoparticles^22^, they too require extensive preparation under harsh conditions (e.g., high temperature and volatile organic solvents) and cannot be readily expanded, thereby limiting their compatibility and development, particularly for sensitive biomedical applications.

Addressing these challenges, we develop a versatile approach, by simultaneous tuning of both nanoparticles and templating frameworks, to prepare diverse architectures with a good spatial control. Named **s**urfactant **t**unable sp**a**tial a**r**chitecture (STAR), this strategy utilizes amphiphilic surfactants to guide nanoparticle arrangement while molding the growing framework. Exploiting the varied interactions of surfactants (polar heads and hydrophobic tails) with MOF constituents and nanoparticles, respectively, the approach regulates framework formation with respect to nanoparticle incorporation, thereby enabling precise spatial control—nanoparticle distribution (central vs. peripheral) and organization (clustered vs. dispersed)—to develop advanced architectures. Importantly, the strategy can be broadly expanded. Through rational selection of surfactants, nanoparticles (and combinations) can be precisely integrated and positioned within various hosts to form different products (e.g., 1D oriented, 3D epitaxial and amorphous). The preparation is fast and safe (< 2 min at room temperature, one-pot synthesis with water as the only solvent), and achieves in situ, templated growth on diverse solid substrates.

Applying the strategy, we develop various STARs for different biotechnology applications. In particular, we prepare a dual-probe STAR for the direct profiling of extracellular vesicle (EV) glycosylation in clinical ascites samples. An attractive circulating biomarker, EVs are nanoscale membrane vesicles (30–200 nm in diameter) actively secreted by cells into the circulation^23–25^. They abound in biofluids and carry reflective molecular cargos (e.g., proteins, nucleic acids, and glycans). To enable direct EV measurements against a complex biological background, we develop the STAR to contain two types of quantum dot probes spatially distributed within the composite to achieve differential responsiveness to biomarkers. We employ this selectivity to enable sensitive and specific detection of EV glycans. As compared to other assemblies, the spatial probe arrangement of STAR achieves robust analysis, even in the presence of interfering agents. When implemented on a miniaturized microfluidic platform, the technology enables rapid and multiplexed clinical analysis of native patient ascites, to reveal glycan signatures of cancer-associated vesicles and differentiate patient prognosis.

## Results

### Surfactant-guided spatial assembly of nanomaterials

**Fig. 1:**
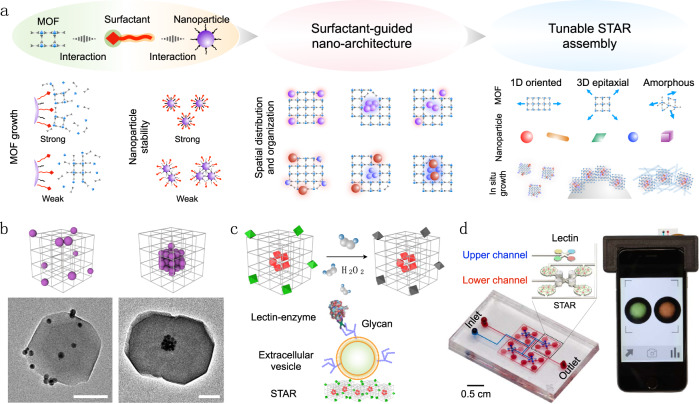
Surfactant-guided spatial assembly of nano-architectures. **a** Schematics of the surfactant-guided nanoparticle integration. The integration performance is determined by surfactant interactions with MOF constituents and nanoparticles. Specifically, strong interaction between surfactants (polar heads) and MOF constituents drives central incorporation and heterogeneous MOF growth around the incorporated nanoparticles, while weak interaction induces tangential integration and homogeneous MOF growth. Likewise, strong interaction between surfactants (hydrophobic tails) and nanoparticles stabilize nanoparticle dispersion while weak interaction leads to nanoparticle aggregation. Through surfactant matching to tune these interactions, the spatial distribution and organization of nanoparticles within MOF hosts can be precisely controlled. The approach is universal for diverse nanoparticles and MOFs, and enables rapid, aqueous synthesis (in solution and in situ on various substrates). **b** Schematics and transmission electron micrographs (TEM) of STAR (Au-ZIF-8) assemblies. Left: Tween 20-guided assembly. Nanoparticles are peripherally dispersed with respect to the MOF host. Right: CTAB-guided assembly. Aggregated nanoparticles are centrally encapsulated within the MOF host. Each experiment was repeated three times independently with similar results. Scale bars, 50 nm. **c** Dual-probe STAR assay for the direct profiling of extracellular vesicle glycans. In the presence of target glycans, the specific binding of lectin-oxidase mediates in situ generation of hydrogen peroxide, which selectively quenches the fluorescence of the peripheral working probes, while leaving the central reference probes in the same assemblies unaffected. **d** Microfluidic device and smartphone-based optical detection platform. The microfluidic device consists of two channels: a lower channel with in situ-grown STARs and an upper channel with preloaded lectins. Solution mixing between the two channels during assay workflow enables specific targeting and signal generation.

Surfactants are amphiphilic and demonstrate distinct interactions with MOFs and nanoparticles, respectively^26,27^. We thus leverage surfactants to mediate and guide nanoparticle integration into MOF hosts to achieve precise control of nanoparticle organization and spatial distribution in STARs (Fig. 1a). Specifically, strong interactions between surfactants (primarily the polar heads) and MOF constituents drive heterogeneous MOF growth around the readily incorporated surfactant molecules; weak interactions favor homogeneous MOF growth and tangential surfactant integration. On the other hand, strong interactions between surfactants (primarily the hydrophobic tails) and nanoparticles stabilize monodispersed nanoparticles while weak interactions induce nanoparticle aggregation. By rational selection of surfactants to tune and mediate these interactions, we design and guide nanoparticle integration in STARs. Figure 1b shows examples of the developed STARs. Through distinct surfactant modifications, gold (Au) nanoparticles are organized monodispersed and peripheral (Fig. 1b, left) or aggregated and central (Fig. 1b, right) with respect to the MOF structure. Importantly, the strategy can be applied to hosts with different crystallinity (e.g., 1D oriented, 3D epitaxial and amorphous) (Supplementary Fig. 1) and various nanoparticles (e.g., material composition, shape, and size) (Supplementary Fig. 2) to develop diverse STARs. The approach not only achieves fast, safe synthesis (<2 min at room temperature, one-pot reaction with water as the only solvent), but also enables in situ templated growth on different solid substrates.

Drawing on these advantages, we developed complementary STARs for various biotechnology applications. In particular, we developed a dual-probe STAR to achieve direct profiling of EV glycans in clinical specimens (Fig. 1c). Using different surfactants, we spatially organized two types of quantum dot probes within the MOF assembly (Supplementary Fig. 2b). Their relative spatial distribution within the MOF host endowed the probes with different responsiveness; probes located peripheral in the formed STAR readily react with external stimuli while probes found central serve as intrinsic references (i.e., peripheral working probes vs. central reference probes). We employed this positional selectivity to achieve sensitive and specific biomarker detection. In the presence of target EV glycan, the specific binding of lectin-oxidase mediates in situ generation of hydrogen peroxide (H_2_O_2_), which selectively quenches the fluorescence of the peripheral working probes, while leaving the central reference probes in the same STAR assemblies unaffected. To streamline the assay workflow, we synthesized the STARs in a miniaturized microfluidic device and performed EV glycan measurements on-chip (Fig. 1d). The device consists of a lower channel, where STARs are grown in situ, and an upper channel, which is preloaded with lectins (Supplementary Fig. 3a). Solution mixing between the two channels is only actuated during assay operation to enable specific EV targeting and signal generation (Supplementary Fig. 3b). Importantly, each chip comprises 16 parallel and independent detection chambers, and can be loaded onto a custom-designed, smartphone-based optical platform for multiplexed fluorescence measurements (Supplementary Fig. 3c).

### Surfactant effects on STAR assembly

**Fig. 2:**
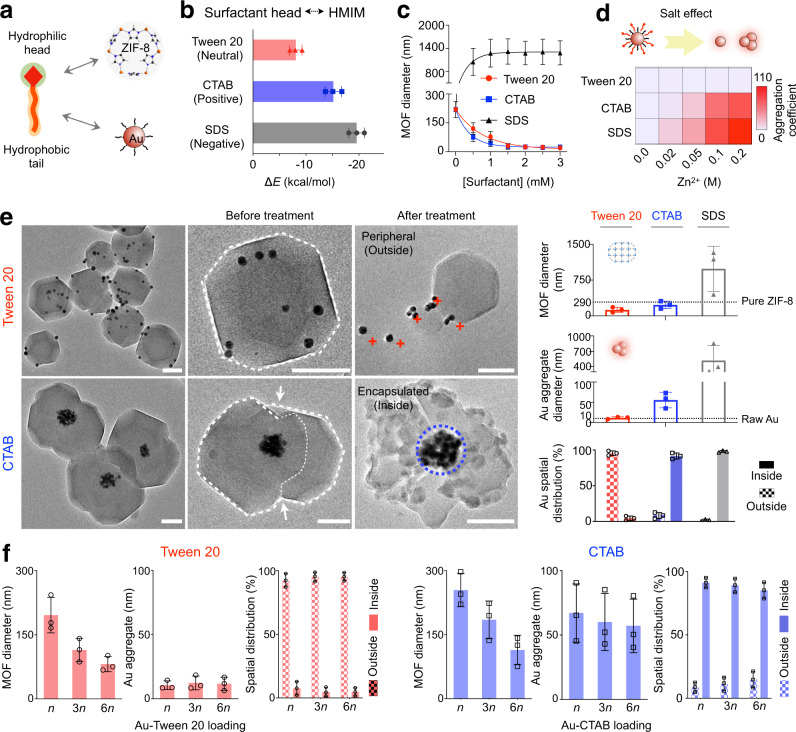
Surfactant effects on STAR assembly. **a** Schematic of surfactant interactions with MOFs and nanoparticles. The surfactant hydrophilic head interacts with MOF constituents (e.g., ZIF-8) and its hydrophobic tail stabilizes nanoparticles (e.g., Au nanospheres). **b** Interaction energies (Δ*E*) between different surfactants and MOF constituents (2-methylimidazole/HMIM), as determined by molecular dynamics simulations. **c** Surfactant effects on MOF growth. MOFs were prepared with increasing surfactant doping. MOF size was monitored through dynamic light scattering characterization. While SDS increased MOF particle size, CTAB and Tween 20 suppressed MOF growth. **d** Surfactant effects on nanoparticle stability. MOF constituents (Zn^2+^) could exert salt effects on surfactants and affect the stability of surfactant-coated nanoparticles. Tween 20 exhibited the strongest stabilization while surfactants with a charged head (CTAB and SDS) showed extensive nanoparticle aggregation. Nanoparticle aggregation coefficient is determined by *d*/*d*_*0*_, where *d*_*0*_ and *d* indicate the nanoparticle diameter before and after the addition of Zn^2+^. **e** Characterization of STARs prepared with different surfactants. Differentially coated Au nanospheres were used to prepare various STARs (Au-ZIF-8). Left: TEM images of various assemblies guided by different surfactants. Dashed lines outline MOF structures and white arrows indicate structural distortions. In situ acid wash (after treatment) verified the peripheral dispersion of Tween 20-coated nanoparticles (red crosses) and the central encapsulation of CTAB-coated nanoparticles (blue circle). Right: corresponding size analyses of MOF host and nanoparticle aggregate as well as quantitative analysis of intra-assembly nanoparticle spatial distribution. Scale bars, 50 nm. **f** Effects of nanoparticle loading concentration. By tuning the loading of Au nanospheres, different-sized STARs with consistent nanoparticle organization and distribution could be obtained. *n* = 5 μg. All measurements were performed in triplicate and the data are displayed as mean ± SD in **b**–**c**, **e**–**f**, and as mean in **d**. Source data are provided as a Source Data file.

In developing the STAR synthesis, we first evaluated the surfactant effects on MOFs and nanoparticles, respectively. Using zeolitic imidazolate framework (ZIF-8) and Au nanospheres as a model system (Fig. 2a), we selected three surfactant species with representative head properties and comparable tail properties, namely Tween 20 (neutral), cetrimonium bromide (CTAB, cationic), and sodium dodecyl sulfate (SDS, anionic). Through molecular dynamics simulations, we profiled the interaction potentials (Δ*E*) of these surfactant heads with MOF constituents (2-methylimidazole/HMIM and Zn^2+^) (Supplementary Fig. 4). The negatively charged SDS demonstrated the strongest interactions with the MOF constituents while the neutral Tween 20 showed the weakest interactions (SDS > CTAB > Tween 20) (Fig. 2b). Experimentally, the addition of SDS substantially increased the size of the prepared MOF, while CTAB and Tween 20 decreased the MOF size, in a dose-dependent and time-progressive manner (Fig. 2c and Supplementary Fig. 5). These empirical results are in agreement with the simulation data, suggesting that SDS as a strong interactor can be readily incorporated into and propagate MOF growth, while weak interactors such as Tween 20 reduce MOF growth. We next evaluated the efficacy of these surfactants in stabilizing nanoparticles. Surfactant-coated Au nanospheres were incubated with an increasing concentration of Zn^2+^ (a ZIF-8 constituent) (Fig. 2d). Among the tested surfactants, Tween 20 offered the strongest stabilization while surfactants with a charged head group (CTAB and SDS) induced extensive nanoparticle aggregation, indicating different surfactant susceptibilities to the salt effects induced by MOF constituents^28^ (Supplementary Fig. 6a–c). For surfactants with a similar head property but different tail lengths (e.g., CTAB vs. DTAB), the longer tail-surfactant provided better nanoparticle stabilization (Supplementary Fig. 6d), likely due to its stronger hydrophobic interaction with nanoparticles^29^.

We next investigated the surfactant effects in preparing different STARs. We prepared Au nanospheres with different surfactant coating before mixing them with MOF constituents (HMIM and Zn^2+^); in all cases, STARs formed rapidly upon reagent mixing (Supplementary Fig. 7a). Transmission electron microscopy (TEM) confirmed architectural differences (e.g., nanoparticle size and distribution) in these STARs (Fig. 2e), consistent with predictions made with respect to various surfactant effects on MOF growth and nanoparticle stability (Supplementary Fig. 7b). Specifically, with Tween 20 coating, monodispersed nanoparticles were peripherally associated with intact MOF hosts; the formed structures showed a small diameter and regular features (outlined by the white dashed line). With CTAB coating, nanoparticle aggregates were encapsulated within the MOF structures; the formed STARs showed an increased diameter and structural distortions (indicated by the white arrows). SDS, on the other hand, resulted in big nanoparticle aggregates encapsulated within large, irregular MOFs (Supplementary Fig. 7c). All nanoparticle spatial distributions within STARs were confirmed through in situ acid washes (Supplementary Fig. 8). While Tween 20-coated nanoparticles could be readily dislodged from the formed STARs (indicated by the red crosses), CTAB-coated nanoparticles remained encapsulated (indicated by the blue circle), even under harsh acid wash conditions. Interestingly, by tuning the nanoparticle loading concentration (Supplementary Fig. 9), different-sized STARs with consistent nanoparticle organization and distribution could be prepared (Fig. 2f). We thus focused all subsequent STAR development using Tween 20 and CTAB as the guiding surfactants.

### Tunable STAR development

**Fig. 3:**
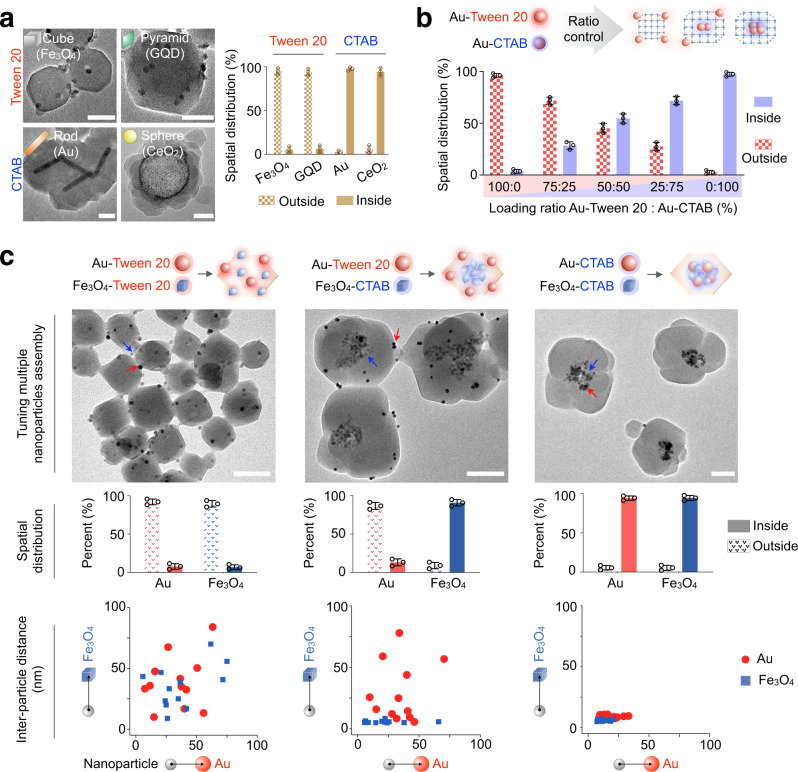
STAR tunability. **a** Different STAR assemblies. Nanoparticles of various shape, size, and material were integrated with ZIF-8 through surfactant guiding. TEM characterization and corresponding analysis of nanoparticle spatial distribution confirmed that all architectures formed abide by the predications as determined by surfactant interactions. Scale bars, 50 nm. **b** Tunable nanoparticle spatial distribution. Through varying the loading ratio of nanoparticle populations (i.e., Au nanospheres coated with Tween 20 and CTAB), precise control of intra-assembly nanoparticle distribution was achieved. The STAR morphology correlated well with the initial nanoparticle loading ratio. **c** Nanoparticle distribution and organization in multi-particle STARs. Using Au nanospheres and Fe_3_O_4_ nanocubes, STARs with distinct architectures were prepared through surfactant matching. Top: TEM images confirming the distinct architectures. Au, red arrow; Fe_3_O_4_, blue arrow. Middle: analysis of nanoparticle distribution with respect to the MOF host. Bottom: analysis of nanoparticle organization with respect to other nanoparticles in the same STAR assemblies. Inter-particle distance was determined through TEM characterization. For each nanoparticle, its average spacing to six nearest Au (*x*-axis) and Fe_3_O_4_ (*y*-axis) particles in the same STAR assembly was plotted. Scale bars, 100 nm. All measurements were performed in triplicate, and the data are displayed as mean ± SD. Source data are provided as a Source Data file.

To evaluate the versatility of the surfactant-guided assembly, we expanded the strategy to prepare different STARs using diverse nanoparticles, of different size, shape, and material, with various MOF hosts. First, using the 3D epitaxial ZIF-8 as a model host, we introduced different surfactant-coated nanoparticles. TEM characterization verified that across all tested nanomaterials, the organization and distribution of nanoparticles within the STARs were consistent with the predicted architectures (Fig. 3a and Supplementary Fig. 10). We further expanded this strategy to integrate nanoparticles in other MOF systems, including another 3D epitaxial MOF (ZIF-67) (Supplementary Fig. 11), 1D oriented MOFs (Cu-BDC, Cu-BTC, Ce-BDC, and Ce-BTC) (Supplementary Fig. 12) as well as amorphous products (Fe-BDC and Fe-BTC) (Supplementary Fig. 13). Systematic characterization of the resultant STARs confirmed that all nanoparticle integration (i.e., spatial organization and distribution) abide by the predications as determined by surfactant interactions.

Inspired by its universality, we exploited the approach to design and develop complex architectures. Using Au nanospheres coated with Tween 20 and CTAB, respectively, we varied the loading ratio of these nanoparticle populations. The approach achieved precise spatial tuning of nanoparticle distribution within individual MOFs; the STAR morphology correlated well with the initial nanoparticle loading ratio and matched closely to the designed architecture (Fig. 3b). Specifically, with increased loading of Tween 20-coated nanoparticles, more particles resided on the MOF periphery; with increased loading of CTAB-coated nanoparticles, more nanoparticle aggregates were observed encapsulated within the MOFs (Supplementary Fig. 14a). We next employed this approach of surfactant matching to precisely integrate and distribute different types of nanoparticles in STARs. Using Au and Fe_3_O_4_ nanoparticles of different size and shape, we coated them with Tween 20 and CTAB to develop multi-particle architectures (Fig. 3c, top and Supplementary Fig. 14b). Nanoparticle spatial distribution (with respect to the MOF host, Fig. 3c, middle) was quantified through in situ acid washes. We further measured inter-nanoparticle distance, through TEM characterization, to reflect nanoparticle organization (with respect to other nanoparticles, Fig. 3c, bottom). The analyses confirmed the efficacy of surfactant matching in tuning particle distribution and organization: (i) with universal Tween 20 coating, all nanoparticles (Au, red; Fe_3_O_4_, blue) were peripherally associated with the MOF host and remained dispersed; (ii) when applied as a mixture, nanoparticles distributed and organized according to their surfactant coating. Tween 20-coated Au nanoparticles (red) were peripherally located and remained dispersed, while CTAB-coated Fe_3_O_4_ nanoparticles (blue) were encapsulated and aggregated within MOFs; (iii) with universal CTAB coating, all nanoparticles were aggregated and located centrally within MOFs.

### Rapid assembly for biotechnology applications

**Fig. 4:**
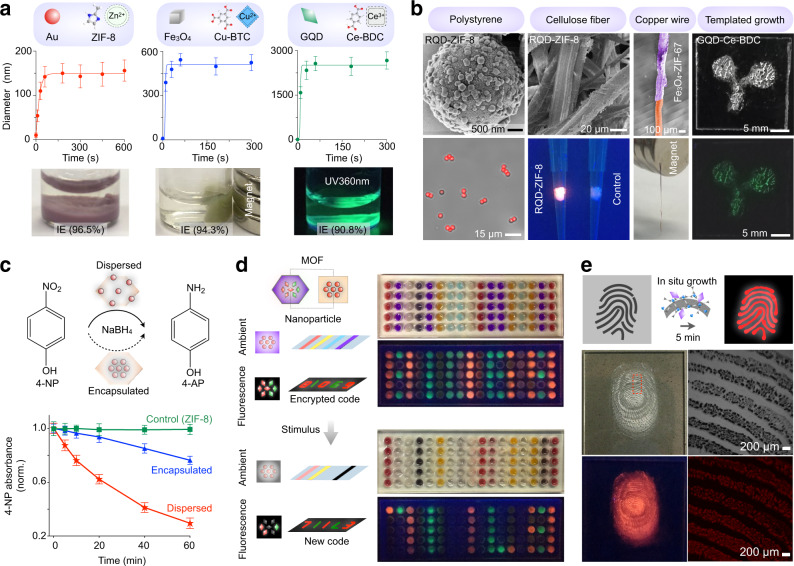
Rapid STAR assembly for biotechnology applications. **a** Rapid assembly of STARs. Using different nanoparticles and MOF hosts, STARs were prepared as a suspension through aqueous synthesis. The reaction was completed in <2 min at room temperature and the formed assemblies presented different properties. The nanoparticle integration efficiency (IE) was determined by measuring the amount of residual nanoparticles in the supernatant. **b** In situ growth of STARs on diverse substrates. Polystyrene beads: scanning electron micrograph (SEM) (top) and fluorescence microscopy image (bottom) of RQD-ZIF-8 grown on beads. Cellulose fiber: SEM (top) and fluorescence microscopy image (bottom) of RQD-ZIF-8 grown on fibers. Copper wire: pseudo-colored SEM image of copper wire carrying Fe_3_O_4_-ZIF-67 (top) and photograph demonstrating its magnetic response (bottom). Templated growth: bright-field (top) and fluorescence (bottom) images of the selective growth of GQD-Ce-BDC; the growth aligned to the seeding pattern as outlined by triglycerides. **c** STARs as nanocatalysts. Using differentially coated Au nanoparticles and ZIF-8 as a host, we prepared STARs with distinct spatial distributions of nanoparticles. The dispersed Au-ZIF-8 demonstrated a much higher catalytic efficiency than the encapsulated version in transforming 4-nitrophenol (4-NP) to 4-aminophenol (4-AP). **d** STARs for data encryption. STARs were prepared as combinations of MOF hosts and surfactant-coated nanoparticles. Due to their material composition, the STARs exhibit different optical properties (e.g., color under ambient lighting and fluorescence under UV excitation); due to their intra-assembly spatial distribution of nanoparticles, the STARs respond differently to external stimuli (e.g., acetic acid) to reveal the encrypted code. **e** Fingerprint detection. Bright-field and fluorescence images of the formed RQD-Ce-BDC along the fingerprint lines. All measurements were performed in triplicate, and the data are displayed as mean ± SD in **a** and **c**. Source data are provided as a Source Data file.

To apply STARs for various biotechnology applications, we first evaluated the robustness of their preparation, in solution as well as on solid substrates. Using different nanoparticles and MOF hosts, we prepared STARs as a suspension through aqueous synthesis (Fig. 4a). The reaction could be completed in <2 min at room temperature, with water as the only solvent. Importantly, the reaction achieved controlled nanoparticle loading and yielded a high nanoparticle integration efficiency (IE > 90%) to form composites with different properties (Supplementary Fig. 15). For composites developed with identical nanoparticles in different MOF hosts, the choice of MOF demonstrates a small influence on the nanoparticle-endowed properties (e.g., fluorescence) but a strong effect on the cellular toxicity of the composites (Supplementary Fig. 16). In addition to inorganic nanoparticles, this aqueous synthesis also enhanced the integration of biological molecules (e.g., proteins and small-molecule drugs) (Supplementary Fig. 17). We further assessed the assembly of STARs on various solid substrates (e.g., polystyrene microspheres, cellulose mesh, copper wire) (Fig. 4b and Supplementary Fig. 18). Through multimodal characterization, we not only observed rapid, in situ STAR formation on different solid supports, but also confirmed its templated growth on treated surfaces, with minimal development in the control regions.

We next applied STARs, prepared in solution or on substrates, for various biotechnology applications. First, using differentially coated Au nanoparticles and ZIF-8 as a host, we prepared STARs with varied spatial distribution of nanoparticles, dispersed vs. encapsulated. We applied these two types of STARs as nanocatalysts for the reduction of 4-nitrophenol (4-NP) to 4-aminophenol (4-AP)^30^ (Fig. 4c). By absorbance measurement, we demonstrated that the dispersed STARs have a higher catalytic efficiency than the encapsulated form (Supplementary Fig. 19). Second, using different combinations of STARs, we developed an encryption array (Fig. 4d). Due to their material composition (e.g., MOFs and nanoparticles), these STARs exhibit different optical properties (e.g., color under ambient lighting and fluorescence under UV excitation); due to their intra-assembly nanoparticle spatial distribution, they respond differently to external stimuli (e.g., acetic acid) to generate different optical signals (Supplementary Fig. 20). By embedding different STARs in a polyacrylamide array (Supplementary Table 1), we encoded information and employed the different responses of STARs (i.e., color and fluorescence changes, in response to stimulus) to reveal the encrypted code. Lastly, we applied the in situ development of STARs to reveal latent fingerprints (Fig. 4e). Motivated by its templated growth along biomolecule-treated surfaces (e.g., lipids, which are commonly found in human fingerprints) (Supplementary Fig. 18b), we leveraged the STAR assembly to achieve rapid and direct visualization of latent fingerprints.

### Identification of glycan signature in clinical samples

**Fig. 5:**
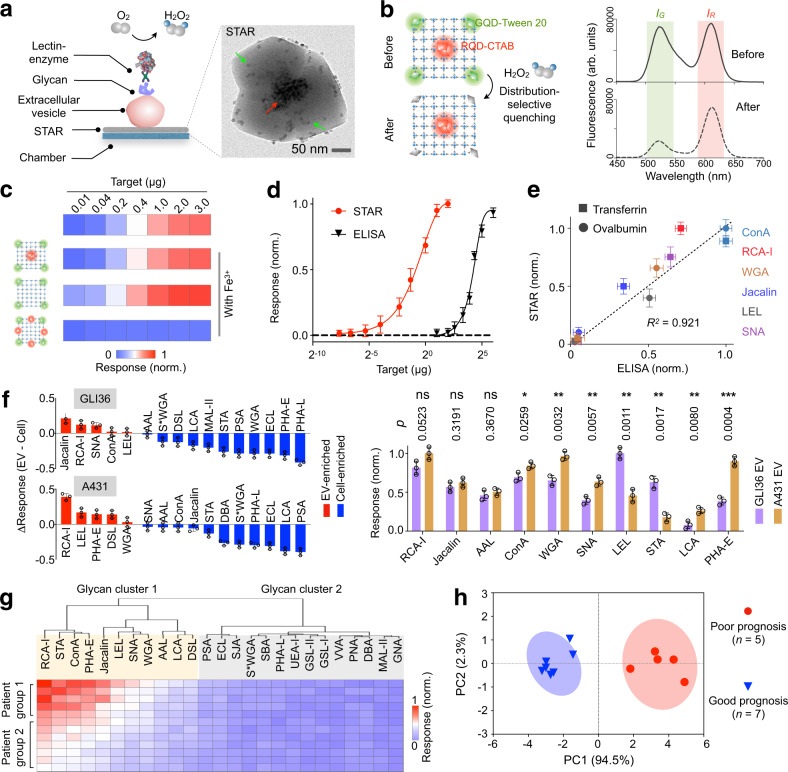
Glycan profiling of extracellular vesicles. **a** Schematic of the STAR glycan assay. A dual-probe STAR architecture was developed on a microfluidic platform. It contained GQDs (green arrow) peripherally dispersed and RQDs (red arrow) centrally encapsulated within the MOF as the working probes and reference probes, respectively. **b** Fluorescence changes. In the presence of target glycan, the specific binding of lectin-oxidase mediates in situ generation of hydrogen peroxide, which selectively quenches the fluorescence of the peripheral working probes, while leaving the central reference probes in the same STARs unaffected. Typical fluorescence spectra showed intensity changes (*I*_G_ and *I*_R_) before and after the assay. **c** Robust performance in the presence of interfering agent. Only the STAR assembly accurately revealed the target glycan concentration, amidst the interfering agent (Fe^3+^, 1 µM). **d** Detection sensitivity of the assay. The limit of detection was assessed by titrating a known quantity of the target glycoprotein (transferrin) and measuring the associated signal response. The detection limit for ELISA was independently assessed based on fluorescence signals. **e** Correlation between STAR and ELISA measurements, when measured with six lectins. *R*^2^, Pearson’s correlation coefficient. **f** Glycan profiling in EVs. Left: comparison of glycan profiles between EVs and their parent cells. ∆Response = Response_EV_ – Response_Cell_. Right: comparison of glycan profiles between EVs derived from different cell origins. All EV measurements were performed with an equal vesicle concentration (5 × 10^8^/ml) through the multiplexed STAR platform. Datasets were normalized to their respective highest. For inter-sample comparisons, multiple pairs of samples were analyzed by two-tailed Student’s *t*-test, and the resulting *p* values were adjusted for multiple hypothesis testing using Bonferroni correction. *P* values are shown and *p* < 0.05 is determined as significant. NS not significant; *p < 0.05, **p < 0.01, ***p < 0.001. **g** STAR analysis of 25 lectin markers in clinical cancer ascites (*n* = 12). Hierarchical clustering of patient profiling data classified the patients into two populations and the glycan expressions into two clusters. **h** Principal component analysis of Cluster 1 glycans for prognosis classification. Ellipses were drawn at 95% confidence. All measurements were performed in triplicate and the data are displayed as mean ± SD in **d**–**f**, and as mean in **g**, **h**. arb. units, arbitrary units. Source data are provided as a Source Data file.

To evaluate its compatibility with clinical samples, we next developed a dual-probe STAR for the direct profiling of EV glycans in clinical biofluids. Specifically, the architecture contained two different types of quantum dots spatially organized within the assembly: red quantum dots (RQD, red arrow) clustered centrally and green quantum dots (GQD, green arrow) associated peripherally as dispersed entities. We grew the dual-probe STARs on a microfluidic platform and utilized their intra-assembly spatial distribution of nanoparticles (and hence different responsiveness) to develop the EV glycan assay (Fig. 5a). EVs were first incubated with the STARs to enable attachment through various interactions (e.g., electrostatic, zinc-carboxylate, and antigen–antibody)^31,32^. In the presence of EV glycan, the specific binding of lectin-oxidase generates hydrogen peroxide in situ: this liberation selectively quenches the fluorescence of the peripheral working probes (GQD), while leaving the central reference probes (RQD) unaffected (Fig. 5b). We thus determined the target glycan signatures by analyzing the relative changes in fluorescence intensities of the two probes. The STARs showed minimal nanoparticle leaching during the assay (Supplementary Fig. 21a), and in the control experiment, demonstrated low fluorescence response in the absence of target glycan (Supplementary Fig. 21b). As compared to other assemblies, the spatial probe arrangement of STAR enabled robust analysis, even in the presence of Fe^3+^ ions^33^, an interfering agent commonly found in clinical samples and known to quench quantum dots (Fig. 5c and Supplementary Fig. 21c). We further determined through a titration analysis that the STAR assay is >500-fold better than the gold-standard enzyme-linked immunosorbent assay (ELISA) assay (Fig. 5d). Importantly, across different glycans measured (Supplementary Table 2), the STAR analysis showed a good correlation (*R*^2^ > 92%) with the ELISA assay (Fig. 5e and Supplementary Fig. 21d). Employing the STAR assay, we next compared the glycan signatures of EVs and their parent cells. Specifically, we measured vesicles derived from brain glial cells (GLI36) and skin epithelial cells (A431) (Supplementary Fig. 22). As compared to their parent cells, EVs were enriched with specific glycans (e.g., RCA-I, LEL). When evaluating vesicles derived from different cell origins, distinct glycan profiles could also be observed (Fig. 5f).

To assess the clinical utility of the STAR assay, we finally conducted a feasibility study using colorectal cancer patient ascites. Using STARs functionalized with antibodies, we developed the assay to achieve direct glycan profiling of cancer-associated EVs in patient specimens. Specifically, we prepared STARs with antibodies against CD24^34,35^, a known cancer antigen, to enrich and measure putative tumor-derived EVs. Multiplexed glycan profiling showed highly varied glycan signatures among the clinical samples (Fig. 5g). Hierarchical clustering of patient profiling data classified the patients into two populations and the glycan expressions into two clusters. This patient classification correlated well with independent clinical evaluation of prognosis based on patient survival data (Supplementary Fig. 23a). We also found that glycan moieties of Cluster 1, but not those of Cluster 2, showed differential measurements across the two patient populations (Supplementary Fig. 23b). Principal component analysis of Cluster 1 glycans enabled good differentiation of patient prognosis (Fig. 5h).

## Discussion

Despite the tremendous interest in developing functional hybrid architectures, this emerging area faces significant challenges. Notably, while the integration of guest nanomaterials with host MOFs present opportunities to design sophisticated architectures, current preparation approaches remain limited with respect to both spatial control as well as integration versatility. To address both challenges, inspired by the amphiphilic nature of surfactants (i.e., polar heads and hydrophobic tails) and their varied interactions with MOF constituents and nanoparticles, respectively, we developed the STAR strategy to enable precise assembly at the nanoscale for diverse biotechnology applications.

With respect to spatial control, the technology uses surfactants to guide nanoparticle integration while molding the growing framework. Unlike conventional approaches which either modify nanoparticles or template framework development, the technology leverages surfactant interactions to tune both simultaneously. Specifically, strong interactions between surfactants and MOF constituents drive central integration and heterogeneous framework growth while weak interactions induce peripheral integration and homogeneous framework; likewise, strong interactions between surfactants and nanoparticles stabilize nanoparticles while weak interactions lead to clustered nanoparticles. The approach is thus programmable and predictable, and yields a high nanoparticle integration efficiency (>90%). It not only tunes the overall morphology but also achieves intra-assembly spatial control—nanoparticle organization and distribution—to develop hybrid architectures.

With respect to integration versatility, the approach can be readily expanded to assemble different nanomaterials with various hosts (e.g., 1D oriented, 3D epitaxial and amorphous). Importantly, unlike conventional synthesis approaches which require harsh chemicals and lengthy processing, the STAR preparation is fast and safe (<2 min aqueous synthesis at room temperature). The technology can therefore be adopted to integrate sensitive, biological molecules (e.g., proteins and small molecule drugs) and achieves in situ growth on different types of solid substrates, to develop various multifunctional assemblies for diverse applications. As an example, we developed a dual-probe STAR platform that uses intra-assembly spatial distribution of nanoparticle probes to achieve differential chemical responsiveness. Employing the technology, we performed direct profiling of vesicle glycosylation in clinical biofluids.

The technology has the potential to be further expanded. Within a single STAR structure, new nanoparticle organizations can be designed and developed. Through rational surfactant matching, different nanomaterials could be precisely positioned with respect to one another within the MOF host. Further developments to regulate the rate of STAR assembly, by ratiometric tuning of MOF constituents or stepwise addition of reactants, are likely to facilitate orderly nanoparticle incorporation. Such developments not only provide a generalizable platform to control and study nanoparticle interactions (e.g., near-field and far-field effects)^36–38^ but also enable the development of multifunctional architectures^39^. Using these STAR assemblies as building blocks, higher-ordered hierarchical architectures can be developed. Through careful structural design, STAR units (and combinations) could be tessellated, using polymers and linkers, to establish sophisticated 2D and 3D superstructures and metamaterials^40,41^; these can dramatically alter the system response, through exotic and cooperative effects that cannot be attained by constituent units alone^42,43^. With its robust synthesis in solution and templated growth on various surfaces, the approach can be readily integrated with other platform technologies. In particular, for biomedical sensing applications, further technical improvements through advanced microfluidics^44^ and array-type patterning^45,46^ could facilitate the application of STAR assemblies for highly-parallel measurements of diverse clinical biomarkers.

## Methods

### Nanomaterial synthesis

All chemicals used for synthesis and modification were purchased from Sigma-Aldrich and used directly, unless otherwise stated. Nanoparticles of diverse size, shape, and material composition were synthesized according to the following methods.

Gold nanosphere (Au NS) was synthesized by rapidly injecting gold precursors into a pre-heated surfactant solution^47^. Briefly, oleylamine (5 ml) was refluxed at 150 °C under nitrogen. A mixture of HAuCl_4_·3H_2_O (0.3 mmol) in oleyamine (1 ml) was rapidly injected into the hot solution and stirred for 1.5 h. The particles were washed by ethanol and dispersed in chloroform for further use.

Gold nanorod (Au NR) was prepared via a seed-mediated growth method^48^. Spherical gold seeds were newly synthesized by vigorously mixing ice-cold NaBH_4_ (0.01 M, 0.6 ml) and an aqueous solution (7.5 ml) containing CTAB (0.1 M) and HAuCl_4_ (0.3 mM). After 5 s mixing, the seed solution was ready for further use. Au NR was synthesized in a water bath at 29 °C. The growth solution was prepared by mixing CTAB (0.2 M, 5 ml), AgNO_3_ (4 mM, 0.25 ml), HAuCl_4_ (1 mM, 5 ml), and ascorbic acid (70 μl, 78.8 mM). The seed solution (12 μl) was then added to the growth solution and allowed to grow overnight. The particles were purified using CTAB^49^ and stored in water for further use.

Iron oxide nanosphere (Fe_3_O_4_ NS) was synthesized according to a published method^50^. Fe-oleate complex was first prepared by reacting metal chlorides and sodium oleate. The Fe-oleate complex (3.6 g) and oleic acid (1.0 g, 90%) were dissolved in 1-octadecene (10 ml, 90%) at room temperature. The reaction mixture was heated to 320 °C for 30 min under nitrogen atmosphere. The particles were precipitated using ethanol, and dispersed in chloroform.

Fe_3_O_4_ nanocube (Fe_3_O_4_ NC) was prepared through thermal decomposition of Fe-oleate precursors^51^. In a typical experiment, 1-octadecene (5 ml) solution containing Fe-oleate (2 g) and sodium oleate (2.2 g, 82%) was heated to 320 °C and refluxed for 60 min to allow nanocube growth. The particles were precipitated using ethanol, and dispersed in chloroform.

CeO_2_ nanosphere (CeO_2_ NS) was prepared through thermal decomposition of cerium nitrate^52^. Briefly, Ce(NO_3_)_3_·6H_2_O (0.108 g, 0.25 mmol) and oleylamine (0.802 g, 3.0 mmol) were dissolved in 1-octadecene (4 ml) at 80 °C. The resultant mixture was then heated to 260 °C for 2 h. The particles were precipitated using ethanol, and dispersed in chloroform.

Silver nanosphere (Ag NS) was prepared by dissolving AgNO_3_ (51 mg, 0.30 mmol) in oleylamine (7.5 mmol, 2.5 ml), which was then injected quickly into refluxing toluene (50 ml)^53^. The reaction was left at reflux overnight, before being cooled. The particles were collected using ethanol, and dispersed in chloroform.

Quantum dot nanopyramid (QD NP) was prepared according to reported methods^54,55^. For the synthesis of blue quantum dot, S precursor solution (0.1 M) was prepared using S powder and 1-octadecene, and Zn precursor solution (0.1 M) was prepared by dissolving ZnO and oleic acid (1:8 molar ratio) in 1-octadecene. To synthesize CdS core, a mixture of CdO (0.2 mmol), oleic acid (1.6 mmol), and 1-octadecene (6 g) was heated to become clear at 260 °C and S precursor solution (1 ml) was rapidly injected. After 30 min, the reaction mixture with formed CdS nanocrystals was cooled to 50 °C. Subsequently, methanol was used to remove unreacted precursors. To grow the ZnS shell, oleylamine (2 ml) was added to the CdS core solution and heated to 120 °C. The Zn and S precursor solutions (1 ml each) were added consecutively. The solution temperature was increased immediately to 220 °C and kept for 20 min to allow the growth of ZnS shell. For the synthesis of GQD and RQD, CdO and zinc acetate were dissolved in oleic acid (5 ml) at 150 °C under nitrogen protection. 1-Octadecene (10 ml) was then added and the reaction temperature was increased to 310 °C. Finally, a stock solution containing trioctylphosphine (3 ml), Se powder, and S powder was quickly injected into the reaction. The reaction was maintained at 310 °C for 10 min before cooling to room temperature. The initial precursor compositions for preparing GQD and RQD were (Cd 0.4 mmol; Zn 4 mmol; Se 0.1 mmol; S 4 mmol) and (Cd 0.4 mmol; Zn 4 mmol; Se 1 mmol; S 2.3 mmol), respectively. The particles were collected and dispersed in chloroform.

### Surfactant modification

To stabilize hydrophobic nanoparticles in aqueous media, surfactant modification was performed through phase transfer reactions. Briefly, the as-synthesized nanoparticles dispersed in organic solvent (0.5 ml, 5 mg/ml) were mixed with aqueous surfactant solutions (0.5 ml). The mixture was sonicated for 3 min and organic solvent was evaporated at 80 °C. The resultant suspension was filtered to remove excess free surfactants.

### Molecular simulation

Molecular dynamics simulations between surfactants and MOF constituents were performed, in a surfactant-constituent pairwise manner, through a commercial software (Materials Studio 2018). We simulated interactions between the surfactant heads and the constituent molecules. For ionic surfactants, interactions were studied with and without counter ions. All molecular structures were modeled by sketch tools and geometrically optimized. The intermolecular interactions were simulated through the Quench task of Forcite module using the COMPASS force field with a microcanonical (NVE) ensemble. For each surfactant-constituent pair examined, three different molecular dynamics simulations were carried out: surfactant–constituent complex, surfactant only, and constituent only. The interaction energy (*E*_s/c_) is calculated as *E*_s/c_ = *E*_s+c_ – (*E*_s_ + *E*_c_), where *E*_s+c_, *E*_s_, and *E*_c_ are the potential energies of surfactant–constituent complex, surfactant only, and constituent only, respectively. All simulations were set with the following parameters: temperature at 25 °C, duration of 50 ps with a time step of 1 fs. Simulation data collected in the last 40 ps were used for structural and statistical analysis.

### STAR preparation

All reactions were performed in water and at room temperature. Surfactant-stabilized nanoparticles (5 mg/ml), metal nodes, including Zn(NO_3_)_2_ (0.05 M), CoCl_2_ (0.05 M), CuSO_4_ (0.025 M), FeCl_3_ (0.025 M), and Ce(NO_3_)_3_ (0.025 M), as well as organic linkers including 2-methylimidazole (HMIM, 2.5 M), benzene-1,4-dicarboxylic acid (BDC, 0.025 M), and benzene-1,3,5-tricarboxylate (BTC, 0.025 M), were used for different STAR assemblies, unless otherwise stated. BDC and BTC were dissolved in water, with the addition of NaOH, and the final solution was kept at pH 6–7. Taking (Au-Tween 20)-(ZIF-8) for example, Tween 20-stabilized Au nanoparticles (2.5 μl) were mixed with Zn(NO_3_)_2_ (50 μl), followed by the addition of HMIM (50 μl). After vigorous mixing, the mixture was allowed to react in static for 10 min. The product was collected through centrifugation and washed by deionized water. All other composites were prepared in a consistent approach.

### Control of nanoparticle distribution in STAR

We utilized different surfactant modifications to tune and direct various nanoparticle assembly and distribution in different MOF hosts. To achieve the desired nanoparticle distribution, we selected respective surfactants, according to their interaction profiles as predicted by the molecular dynamics simulations. For the integration of a single type of nanoparticles, we regulated the loading ratio of different surfactant-modified nanoparticles to control their spatial distributions. For example, to prepare Au-ZIF-8, Au-Tween 20 and Au-CTAB (2 μl, 5 mg/ml) were independently prepared, and mixed with Zn^2+^ solution (50 μl, 0.05 M). The solutions were added in various proportions to HMIM (50 μl, 2.5 M). After vigorous mixing, the reaction was allowed to react in static for 10 min. The prepared composites were collected through centrifugation and characterized for spatial distribution. For the integration of multiple types of nanoparticles, to achieve the desired spatial distribution, we matched the nanoparticles with different surfactant modifications. For example, to prepare (Au, Fe_3_O_4_)-ZIF-8 composite with monodispersed Au locating peripherally (outside) and aggregated Fe_3_O_4_ encapsulated centrally (inside) of the MOF host, Au-Tween 20 and Fe_3_O_4_-CTAB (2 μl, 5 mg/ml) were independently prepared, mixed with Zn^2+^ solution (50 μl, 0.05 M), and added in various proportions to HMIM solution (50 μl, 2.5 M). After vigorous mixing, the reaction was allowed to react in static for 10 min. The prepared composites were collected through centrifugation and characterized for spatial distribution.

### STAR characterization

Dynamic light scattering analysis of particle diameter and zeta potential was performed with Zetasizer Nano ZS instrument (Malvern). Bright-field and fluorescence images were acquired on an inverted microscope (Leica MDi8, Leica application suite X 3.6.123246). Powder X-ray diffraction was performed in the 2*θ* range 5–50° at a scanning rate of 2° min^−1^ on an X-ray diffractometer (Bruker D8 Advanced) with a Cu-*K*α radiation at 40 kV and 40 mA. For scanning electron microscopy analysis, samples were loaded on silicon slides, sputter-coated with gold (Leica) before being examined (JEOL 6701). For TEM analysis, samples were loaded onto carbon-coated copper grids (Latech) for imaging (JEOL 2010F). To examine the spatial distribution of nanoparticles in MOF host, the prepared composites were washed in situ on TEM grid. Specifically, STAR solution (10 μl) was first deposited on a TEM grid for 5 min. Following gentle removal of the suspension, wash buffers of various pH (10 μl) was dropped and incubated on the grid, before being wicked away with a piece of filter paper. The prepared grid was finally dried for further TEM analysis to evaluate the remaining nanoparticle distribution and morphology in MOF structures, so as to optimize the wash conditions. To evaluate cellular toxicity, we employed the MTS cell proliferation assay (Thermo Scientific). Per manufacturer’s protocol, epithelial cells (A431) were seeded and incubated with different concentrations of STAR composites for 24 h. After the addition of MTS reagent, absorbance (490 nm) was measured to evaluate cell viability (Tecan, SparkControl v2.1).

### Analysis of nanoparticle spatial distribution

To quantify nanoparticle concentration in the as-synthesized STAR assembly (*N*_total_), we measured its elemental content by inductively coupled plasma-optical emission spectrometry (ICP-OES) (Perkin Elmer Avio 500). The nanoparticle spatial distribution in MOF was evaluated through in situ washes, as described above. As the wash buffer infiltrates the MOF structure, it first dislodges the peripherally associated nanoparticles (outside) from the MOF host; the centrally encapsulated nanoparticles (inside) remain within the MOF host. The wash conditions were optimized through TEM characterization to achieve clear differentiation of the outside population: acidic HCl buffers were used for HMIM-based and BTC-based samples, and alkaline NaOH buffers were used for BDC-based samples. All wash incubations were kept to 5 min. To quantify nanoparticles dislodged into the supernatant (*N*_outside_), after in situ washes, we recovered the supernatant and quantified its elemental content through ICP-OES.

The nanoparticle spatial distribution in STAR assembly is determined as below

1 $${\rho }_{{\rm{outside}}}={N}_{{\rm{outside}}}/{N}_{{\rm{total}}}$$

2 $${\rho }_{{\rm{inside}}}=1\,{-}\,{\rho }_{{\rm{outside}}}$$

where *ρ*_outside_ and *ρ*_inside_ are fractions of nanoparticles distributed outside and inside of the STAR assembly, respectively. *N*_total_ is the total number of nanoparticles in the STAR assembly. *N*_outside_ is the number of peripherally associated nanoparticles, dislodged into the supernatant after in situ washes.

### In situ growth on substrates

STAR assemblies were grown in situ on various substrates at room temperature. Briefly, surfactant-modified nanoparticles, metal node solution, and organic linker solution were mixed and loaded immediately onto substrates (e.g., polystyrene, metal, cellulose, glass). After 10 min incubation, the substrates were washed in water to remove unbound composites and dried for further characterization. The synthesis precursors for different substrates are as follows: polystyrene beads: Zn^2+^ node (50 mM, 0.5 ml), HMIM (2.5 M, 0.5 ml), RQD-Tween 20 (5 mg/ml, 25 μl); copper wire: Co^2+^ node (50 mM, 0.5 ml), HMIM (2.5 M, 0.5 ml), Fe_3_O_4_ nanocube-SDS (5 mg/ml, 25 μl); cellulose fiber: Zn^2+^ node (25 mM, 0.5 ml), HMIM (2.5 M, 0.5 ml), RQD-Tween 20 (5 mg/ml, 25 μl); glass slide: Ce^3+^ node (25 mM, 0.5 ml), BDC (25 mM, 0.5 ml), GQD-Tween 20 (5 mg/ml, 25 μl).

### STAR for catalysis

Two Au-ZIF-8 composites with distinct Au nanoparticle distributions (i.e., peripherally dispersed Au-Tween 20 vs. encapsulated Au-CTAB) were adopted as catalysts for the conversion of 4-nitrophenol (4-NP) to 4-aminophenol (4-AP) in the presence of reductive NaBH_4_. The Au-ZIF-8 composites were synthesized using Au nanoparticles (2.5 μl, 5 mg/ml), Zn(NO_3_)_2_ (50 μl, 0.05 M), and HMIM (50 μl, 2.5 M). For the analysis of catalytic efficiency, 4-NP (0.25 ml, 1 mM, pH = 10) and freshly prepared aqueous NaBH_4_ (0.25 ml, 50 mM) were mixed in 3.5 ml of water. Subsequently, Au-ZIF-8 composites (0.1 ml, 5 mg/ml) were added to the reaction. UV-Vis absorption spectra were recorded in real time to monitor the concentration of 4-NP (Tecan, SparkControl v2.1).

### STAR for encryption

A poly(methyl methacrylate) (PMMA) array with 5 × 17 wells was prepared via a tabletop CO_2_ laser engraver (Universal Laser Systems). We employed microscopic (STAR assemblies) and macroscopic patterning (well positioning) to encrypt information (Supplementary Table 1). For microscopic encryption, various STAR assemblies were synthesized; these assemblies contain combinations of nanoparticles, differentially distributed in various MOF hosts. For macroscopic patterning, different STAR assemblies were mixed with polyacrylamide gel precursor (4% PAGE gel, Bio-Rad) and allowed to polymerize in defined wells. To achieve information transformation, the device was treated with 2% acetic acid as the stimulus for 10 min, before being washed in water, to reveal the encrypted code.

### STAR for fingerprint detection

To reveal latent fingerprints on a solid surface, MOF constituents (Ce^3+^ and BDC) and quantum dots (RQD) stabilized by Tween 20 were applied immediately onto the surface. Specifically, a reaction mixture comprising Ce^3+^ node (25 mM), BDC (25 mM), and RQD-Tween 20 (5 mg/ml), mixed in a volumetric ratio of 100:100:1, was applied to the surface. After a quick incubation (5 min at room temperature), the unbound materials were flushed with water. The fingerprint-induced STAR patterning could be visualized under UV excitation (365 nm).

### EV isolation and characterization

EVs derived from human brain glial cells (GLI36) and skin epithelial (A431) were collected through gradient centrifugation. Cells were cultured in Dulbecco’s modified Eagle’s medium (HyClone) supplemented with 5% vesicle-depleted fetal bovine serum (dFBS, HyClone) and penicillin–streptomycin (Corning). The culture medium was filtered through a 0.8-μm membrane filter (Millipore) and pelleted at 10,000*g* for 20 min to deplete cell debris. The supernatant was centrifuged at 100,000*g* for 2 h to concentrate EVs. Collected EVs were analyzed through nanoparticle tracking analysis (NTA) system (NS300, Nanosight NTA v3.3) to quantify their size distribution and concentration. All NTA measurements were performed with identical system settings, with ~50 vesicles in the field of view to achieve optimal counting. For TEM analysis of EVs, samples were fixed with 2% paraformaldehyde, loaded onto a copper grid (Latech), and contrast-stained with uranyl oxalate and methyl cellulose mixture before TEM analysis (JEOL 2010F).

### STAR assay for glycosylation profiling

Operation steps are illustrated in Supplementary Fig. 3b. In a typical procedure, 5 μl of sample was introduced into the detection chambers. After 5 min incubation, the chambers were blocked by BSA (2% w/v) for 5 min. PBS buffer was introduced to flow through the top chambers, dissolving the preloaded lectins. The lectin solution was further driven into the bottom detection chambers, allowing the biotinylated lectins to bind to target glycan moieties for 5 min. Next, streptavidin-conjugated glucose oxidase (GOD) solution (10 μg/ml) was introduced into the detection chambers and incubated for 5 min. Glucose solution (2 mg/ml) was then added to enable GOD oxidation of glucose to generate hydrogen peroxide (H_2_O_2_); this liberated H_2_O_2_, produced only in the presence of specific bound lectins, selectively quenches peripherally distributed GQD (outside) in the MOF host, but does not affect the centrally encapsulated RQD (inside). After a 10 min reaction, fluorescence measurements were performed to profile glycosylation.

All fluorescence signals are calculated relative to the central reference population (RQD):

3 $$S={I}_{\mathrm{G}}/{I}_{\mathrm{R}}$$

where *I*_G_ and *I*_R_ are the fluorescence intensities of GQD and RQD, respectively.

For glycosylation analysis, the signal response is determined as below

4 $$R=1\,{-}\,({S}_{\mathrm{s}}/{S}_{\mathrm{o}})$$

where *S*_o_ and *S*_s_ are the fluorescence signals before and after sample incubation, respectively.

### Microfluidic platform fabrication

A prototype dual-layer microfluidic device (Supplementary Fig. 3a) was fabricated through standard soft lithography. Briefly, SU8-negative photoresist (SU8-2025, Microchem) was used to prepare the cast molds. The photoresist was spin-coated onto a silicon wafer and patterned with photomasks using a cleanroom mask aligner (SUSS MicroTec). After UV light exposure, the cast molds were developed under agitation. The molds were chemically treated with trichlorosilane vapor inside a desiccator, and then a mixture of polydimethylsiloxane (PDMS, Dow Corning) and crosslinker at a ratio of 10:1 was casted onto the molds and cured at 80 °C. The obtained PDMS replicas and glass substrate were aligned and surface bonded together after the plasma treatment, producing the microchannel.

To enable in situ STAR growth in the bottom detection chamber, 5 μl of synthesis solution comprising Zn^2+^ (25 mM), HMIM (1.25 M), GQD-Tween 20 (0.5 mg/ml), and RQD-CTAB (0.5 mg/ml) was introduced into the device. After 10 min incubation, the device was flushed with water to remove unbound composites. To confine lectins to the top chambers, lectins were dissolved in PBS (15 μg/ml, Vector Laboratories) with excipient (PEG 2000, 1 mg/ml), preloaded into defined chambers of the upper channel layer and lyophilized before PDMS bonding.

### Smartphone-based sensor

To enable smartphone-based evaluation of the STAR assay, we developed a sensor consisting of five components (Supplementary Fig. 3c): a 3D-printed optical cartridge, a UV LED source, a light diffuser, two optical filters, and a magnification lens. The optical cartridge was fabricated using a UV-curable resin (HTM 140) by a desktop 3D printer (EnvisionTEC, Aureus). The light source (Chaoziran S&T) was customized with a UV LED diode with a central wavelength at 365 nm and the emitted UV light was spread homogeneously by a light diffuser (Thorlabs DG05). Two bandpass filters with center wavelengths of 520 and 610 nm were used for measuring fluorescence of GQD and RQD, respectively. The magnification lens (Thorlabs LA4280) was set before the smartphone camera to improve the image quality. The assembled system having dimensions 32 mm (width) × 88 mm (length) × 35 mm (height) was equipped with a sliding slot for quick attachment to the smartphone. The fluorescence images was processed by ImageJ (v1.53) to obtain the fluorescence intensity.

### ELISA assay

Samples were adsorbed onto ELISA plates (Thermo Scientific) and blocked using PBS containing 1% BSA. After washing, biotinylated lectins (5 μg/ml, Vector Laboratories) were introduced in PBS containing 1% BSA. Following incubation (1 h at room temperature), streptavidin-conjugated GQD-ZIF-8 probes were added. Fluorescence signal was determined through a commercial plate-reader (Tecan, SparkControl v2.1).

### Clinical measurements

The study was approved by the National University Hospital (2016/01088) and SingHealth (2015/2479) Institutional Review Boards. All subjects were recruited according to IRB-approved protocols after obtaining informed consent. Ascites samples were collected from colorectal cancer patients, centrifuged at 500*g* for 10 min, and filtered through a 0.8-μm membrane filter (Millipore). All samples were de-identified and stored at -80 °C before glycan analysis.

For clinical analysis, ascites samples were used directly. To enable selective measurement of glycan signatures on EVs, we first functionalized STAR assemblies with antibodies against CD24 (eBioscience), through electrostatic attraction and thiol-zinc affinity. Following antibody modification and subsequent BSA blocking, ascites samples (5 μl) were introduced for direct analysis, as described above. All STAR measurements were performed relative to respective sample-matched, no-lectin control. Clinical evaluation of patient characteristics was determined independently. Specifically, patient prognosis was determined by the overall survival from the time of collection of ascites. Patients were deemed to have a good prognosis when the overall survival was more than ten months. Conversely, patients were determined to have a poor prognosis if the overall survival was less than 5 months. All STAR measurements were performed blinded from these clinical evaluations.

### Statistical analysis

All measurements were performed in triplicate, and the data are displayed as mean ± standard deviation. Correlations were performed with linear regression to determine the goodness of fit (Pearson’s correlation coefficient, *R*^2^). For inter-sample comparisons, multiple pairs of samples were analyzed by two-tailed Student’s *t*-test, and the resulting *p* values were adjusted for multiple hypothesis testing using Bonferroni correction. For unsupervised hierarchical clustering analysis, STAR profiling of glycan signatures were clustered using Euclidean distance metric and complete linkage (Morpheus, Broad Institute, v0.1.1.1). The lectin markers were grouped into two clusters, according to patient expression profiles. Principal component analysis was performed using Minitab (v.19.2020) based on a combination of significant lectin markers to categorize the patient samples according to their clinical prognosis. All other statistical analyses were performed using GraphPad Prism (v.7.0c).

### Reporting summary

Further information on research design is available in the Nature Research Reporting Summary linked to this article.

## Supplementary information

Supplementary Information

Reporting Summary

## Data Availability

All data supporting the findings of this study are included within the article and its supplementary information, and are also available from the corresponding author upon reasonable request. Source data are provided with this paper.

## Source data

Source Data
