# Supplementary material for: Surfactant-guided spatial assembly of nano-architectures for molecular profiling of extracellular vesicles
Source: Nat Commun. 2021 Jun 30;12:4039. doi: 10.1038/s41467-021-23759-9 (PMC8245598; doi:10.1038/s41467-021-23759-9)
Supplement: Supplementary file 1 — Supplementary Information [file 41467_2021_23759_MOESM1_ESM.pdf]

SUPPLEMENTARY INFORMATION

Surfactant-guided spatial assembly of nano-architectures for molecular profiling of extracellular vesicles

Zhigang Wang[1], Haitao Zhao[1], Yan Zhang[1,2], Auginia Natalia[1,2], Chin-Ann J. Ong[3], Melissa C.C. Teo[3], Jimmy B.Y. So[4,5], Huilin Shao[1,2,4,6,*]

[1] Institute for Health Innovation & Technology, National University of Singapore, 117599, Singapore
[2] Department of Biomedical Engineering, Faculty of Engineering, National University of Singapore, 117583, Singapore
[3] Division of Surgical Oncology, National Cancer Centre, 169610, Singapore
[4] Department of Surgery, Yong Loo Lin School of Medicine, National University of Singapore, 117597, Singapore
[5] Division of Surgical Oncology, National University Cancer Institute, 169610, Singapore
[6] Institute of Molecular and Cell Biology, Agency for Science, Technology and Research, 138673, Singapore

* Corresponding author
Huilin Shao, PhD
National University of Singapore
MD6, 14 Medical Drive
#14-01, Singapore 117599
(65) 6601 5885
huilin.shao@nus.edu.sg

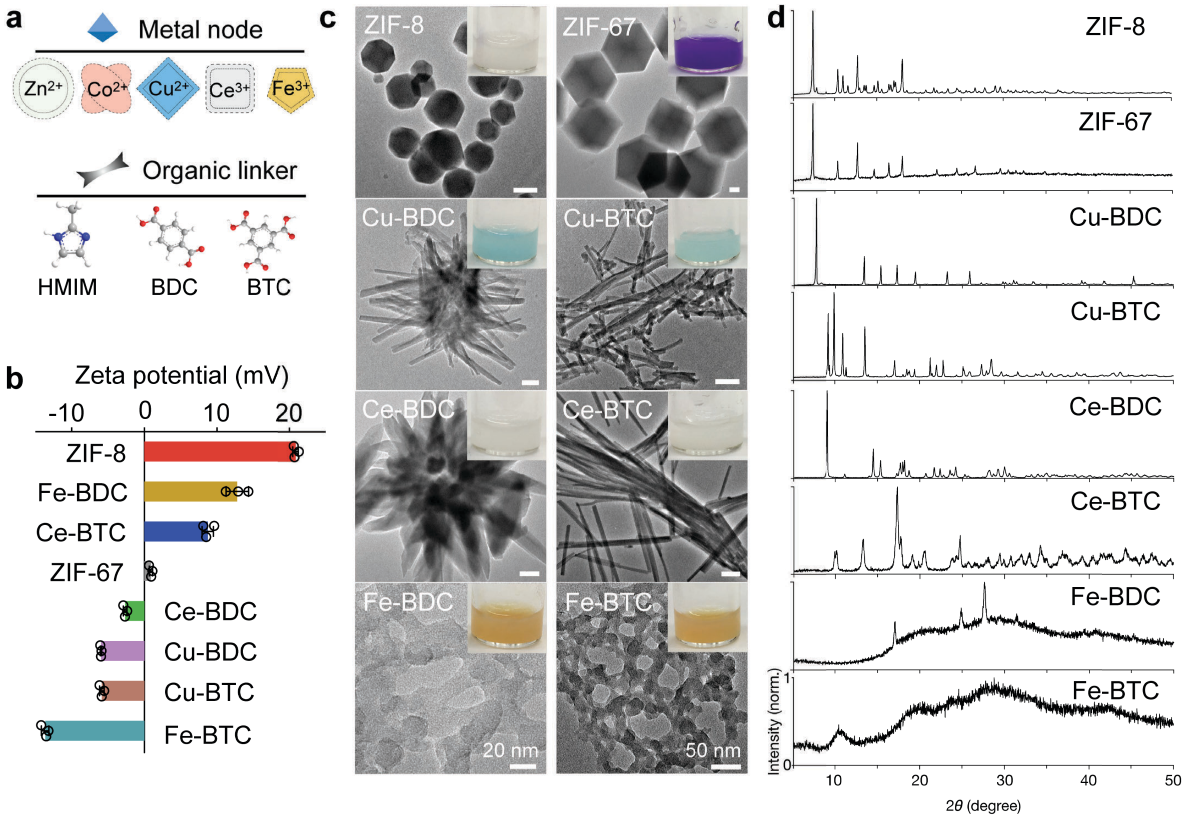

Supplementary Fig. 1: Characterization of different MOFs.

a Schematic of metal nodes and organic linkers. **b** Zeta potential of MOFs measured in water. **c** Photographs (insert) of aqueous MOF suspensions with different optical properties, and TEM images of MOFs displaying various morphologies, including 3D epitaxial ZIF-8 and ZIF-67, 1D-oriented Cu-BDC, Cu-BTC, Ce-BDC and Ce-BTC, and amorphous Fe-BDC and Fe-BTC. Each TEM experiment was repeated three times independently with similar results. **d** Powder X-ray diffraction (PXRD) analysis confirmed the high crystallinity of the 3D epitaxial MOFs (ZIF-8, ZIF-67) and 1D oriented MOFs (Cu-BDC, Cu-BTC, Ce-BDC, Ce-BTC) and the weak crystallinity of the amorphous products (Fe-BDC, Fe-BTC). All measurements were performed in triplicate and the data are displayed as mean ± SD in **b**. All unlabeled scale bars, 200 nm. Source data are provided as a Source Data file.

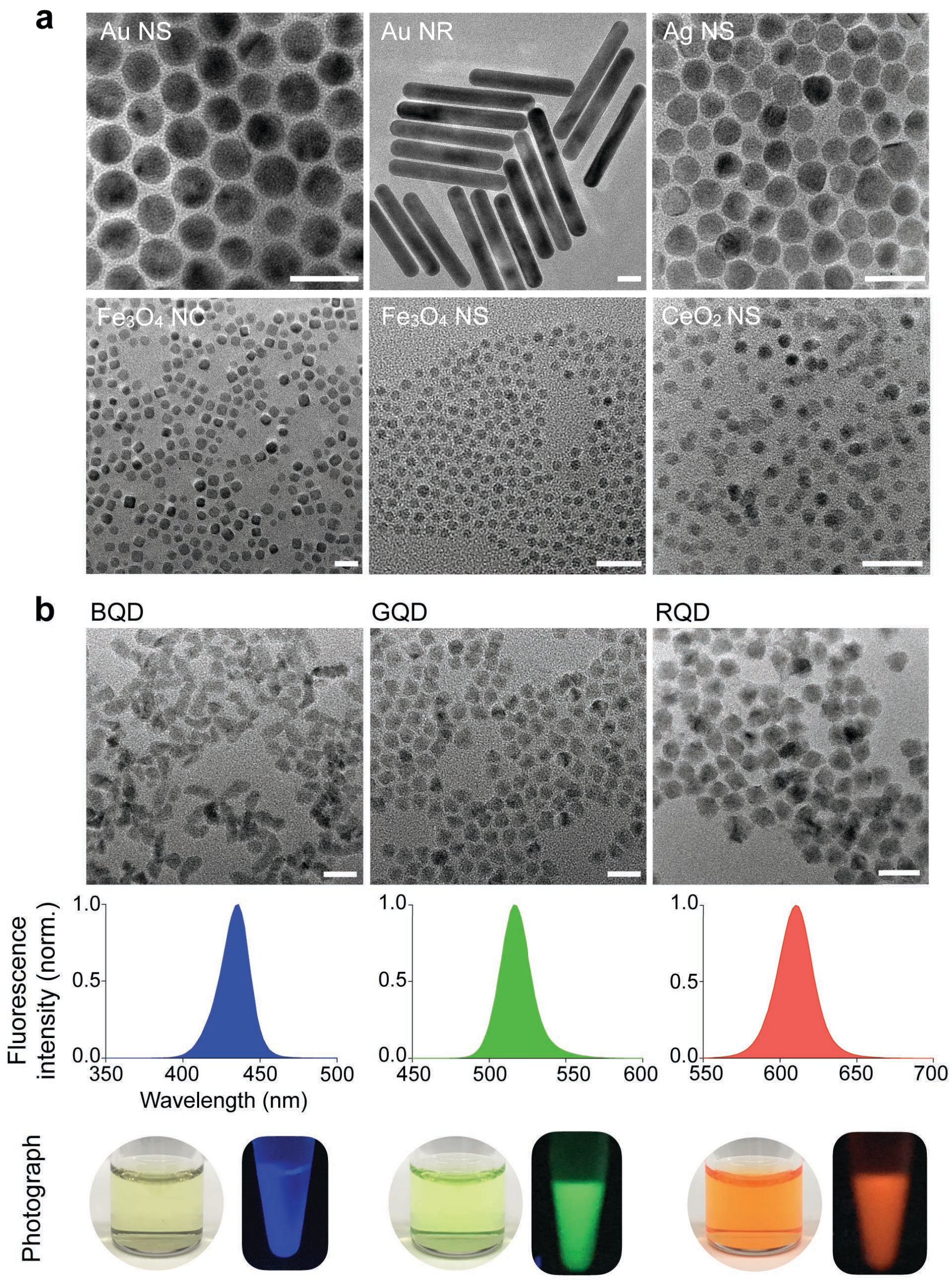

Supplementary Fig. 2: Characterization of different nanoparticles.

a TEM images of nanoparticles, including gold nanosphere and nanorod (Au NS, Au NR), silver nanosphere (Ag NS), magnetic nanocube and nanosphere (Fe_3O_4 NC, Fe_3O_4 NS), cerium dioxide nanosphere (CeO_2 NS). **b** Quantum dots emitting blue, green and red fluorescence respectively (BQD, GQD, RQD). Emission spectra of the quantum dots were measured under UV excitation (365 nm). Each TEM experiment was repeated three times independently with similar results. Scale bars, 20 nm. Source data are provided as a Source Data file.

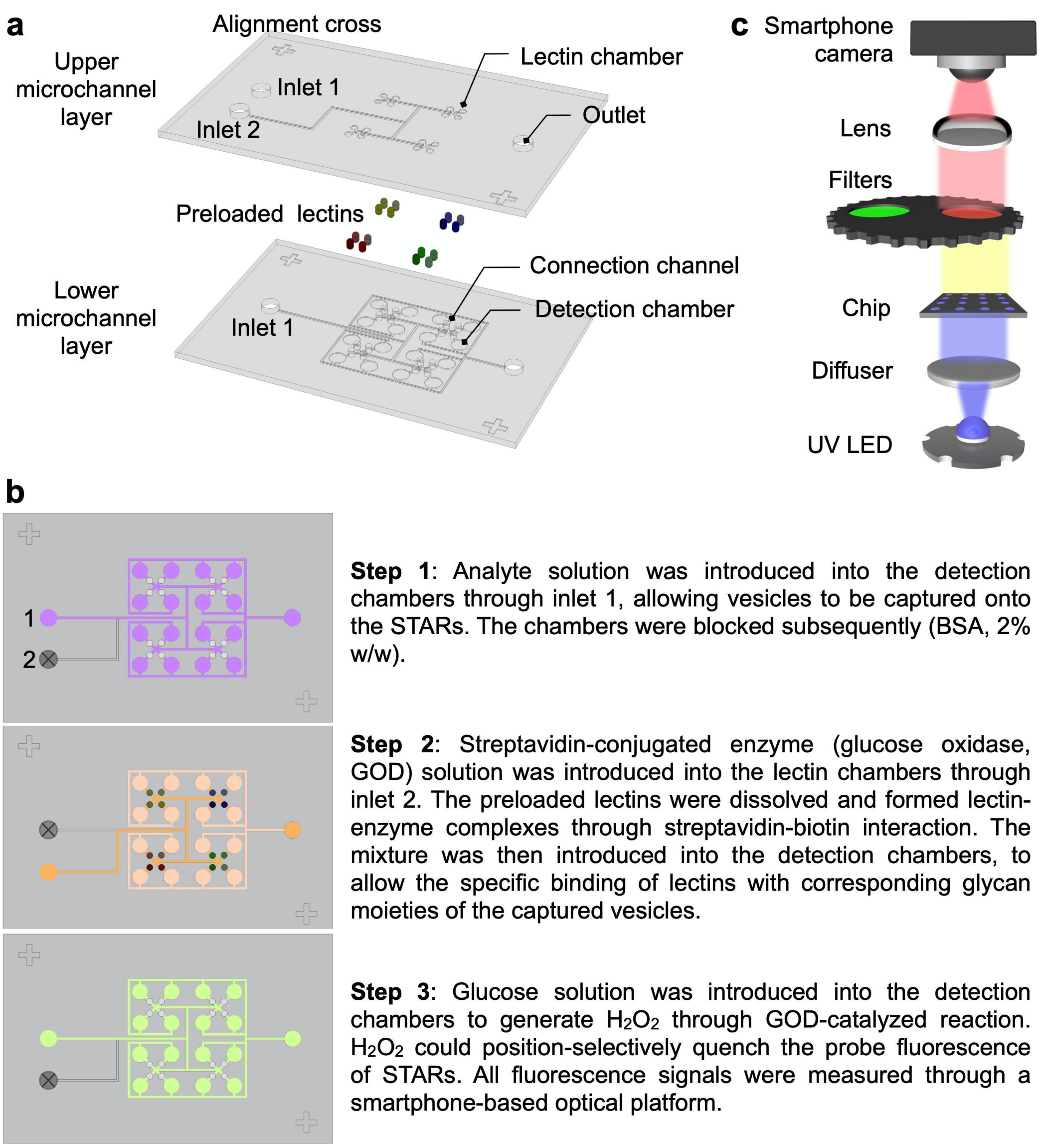

Supplementary Fig. 3: Smartphone-based STAR microfluidic assay.
a Exploded schematic of the STAR assay cassette. The cassette comprises an upper microfluidic channel with preloaded lectins in the lectin chambers and a lower microfluidic channel with dual-probe STAR in the detection chambers. The two layers are interconnected though the connection channels. **b** Operation of the microfluidic platform. **c** Schematic of the smartphone-based detection system. Different fluorescence measurements could be performed through varying the filter configuration.

a

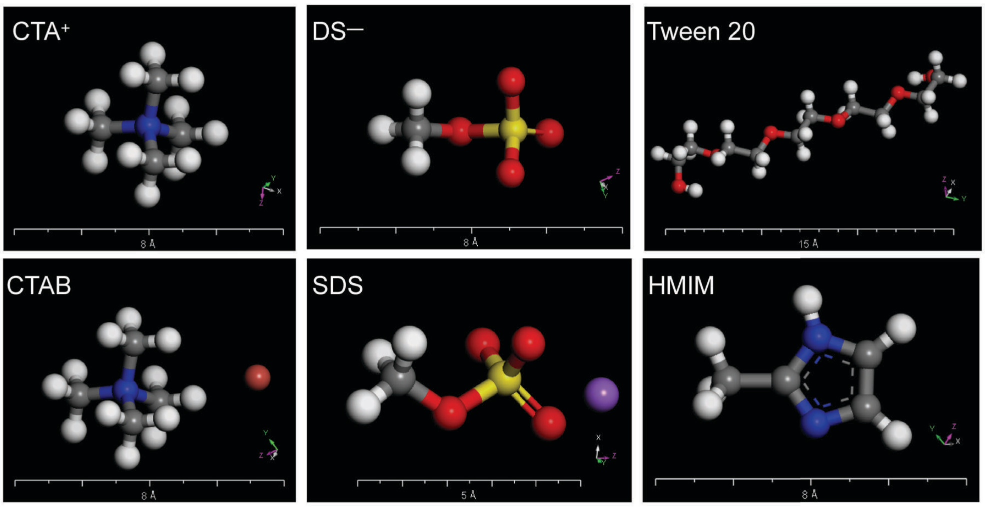

b

$\Delta E_{s/p}$	CTA^+	CTAB	DS^-	SDS	Tween 20
HMIM	-12.05	-15.06	-16.92	-19.57	-7.97
Zn^{2+}	-0.25	-0.26	-0.17	-0.1	3.15

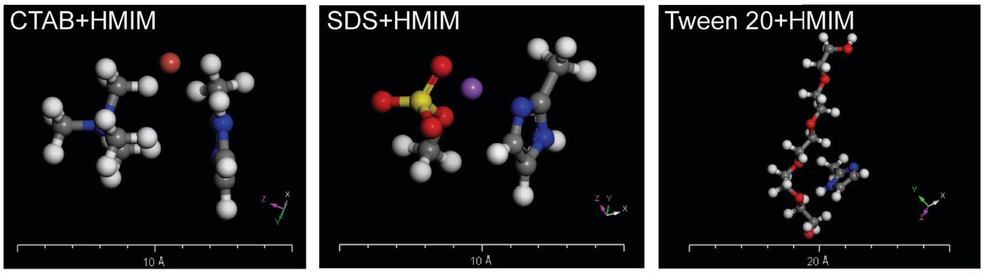

Supplementary Fig. 4: Molecular dynamics simulation.

a Molecular structures of the surfactant heads and ZIF-8 constituents. **b** Top: potential energies (ΔE) computed from molecular dynamics simulations. Bottom: simulation snapshots. For ionic surfactants, interactions were studied with and without counter ions. All simulations were set with the following parameters: temperature at 25 °C, duration of 50 ps with a time step of 1 fs. Simulation data collected in the last 40 ps were used for structural and statistical analysis. C atom, grey; H atom, white; N atom, blue; O atom, red; S atom, yellow; Na atom, purple; Br atom, brick red.

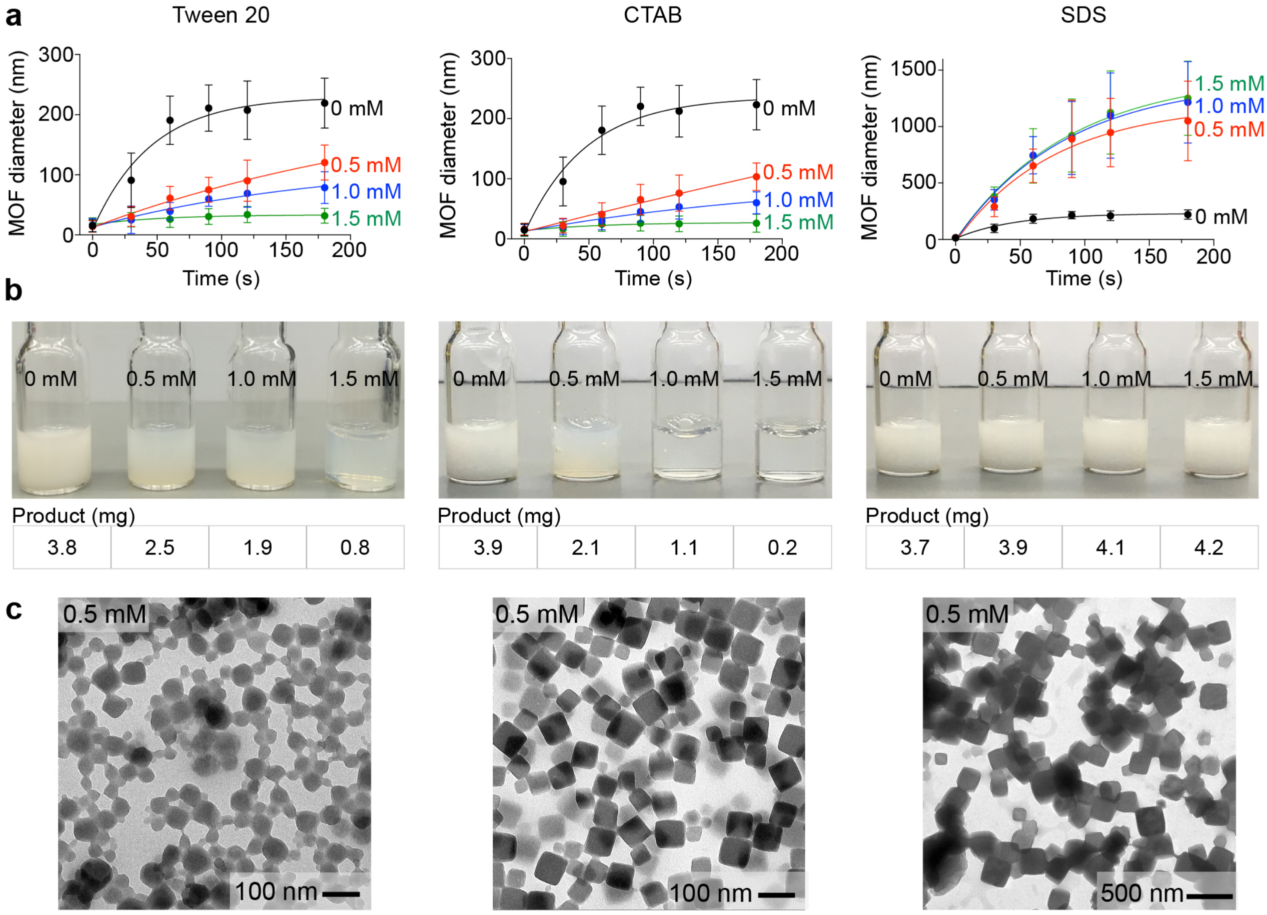

Supplementary Fig. 5: Surfactant effects on ZIF-8 growth.

a Surfactant effects on MOF diameter. ZIF-8 particles were grown in the presence of various surfactants and the particle diameter was determined through dynamic light scattering analysis in real time. **b** Photographs of MOF suspensions prepared at four surfactant concentrations. For Tween 20 and CTAB samples, the opacity of the suspensions decreased with increasing surfactant loading, consistent with decreased MOF production in the suspensions. **c** TEM images of MOF products prepared with respective surfactants (0.5 mM). Each TEM experiment was repeated three times independently with similar results. All measurements were performed in triplicate and the data are displayed as mean ± SD in **a**. Source data are provided as a Source Data file.

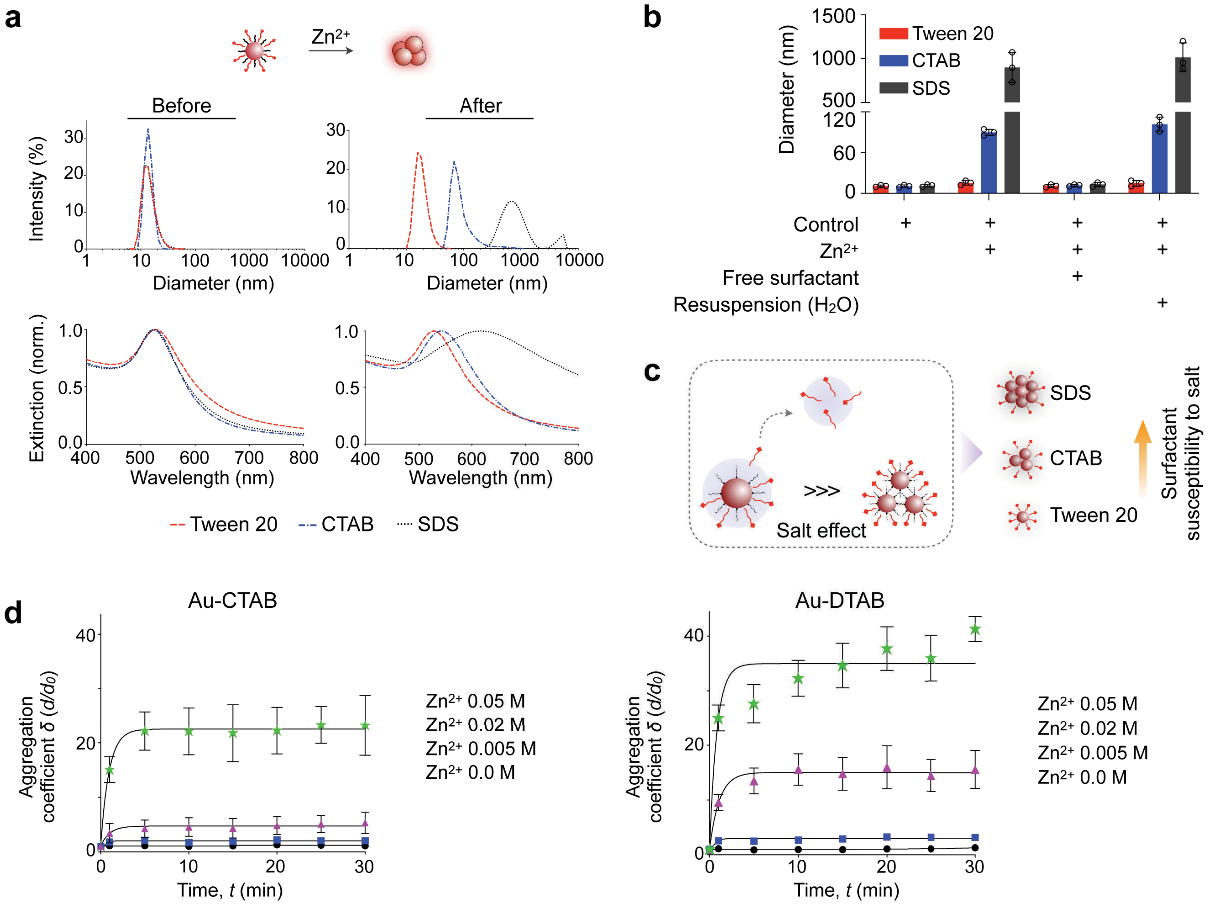

Supplementary Fig. 6: Salt-induced aggregation of Au nanospheres.

a Diameter distribution (upper) and UV-Vis absorbance (lower) of Au nanospheres before and after the addition of Zn^{2+}. The increased diameter and red-shift in absorbance peak verified the extent of Au nanosphere aggregation (SDS > CTAB > Tween 20). **b** Average diameter of Au nanospheres under different conditions. Free surfactants could inhibit the aggregation. The resuspension in H_2O could not reverse the Au aggregation. Au nanospheres were used at 1 mg/ml, in the presence of 0.05 M of Zn^{2+}. **c** Schematic of Au nanosphere aggregation induced by MOF constituents. Salt can decrease the critical micelle concentration of surfactants by decreasing the electrostatic repulsion between the surfactant head groups[1], leading to aggregation of surfactant-coated nanoparticles. Surfactants have different susceptibilities to salts[2], resulting in distinct aggregation propensities. **d** Surfactant stabilization effects. Au nanospheres were coated with surfactants of identical head groups and different tail lengths (CTAB and DTAB). The longer-tailed surfactant offered a stronger stabilization capability in reducing nanoparticle aggregation. Aggregation coefficient (δ) is determined by d_t/d_0, where d_0 and d_t refer to the average diameters of Au nanospheres at the initial and time t, respectively. All measurements were performed in triplicate and the data are displayed as mean ± SD. Source data are provided as a Source Data file.

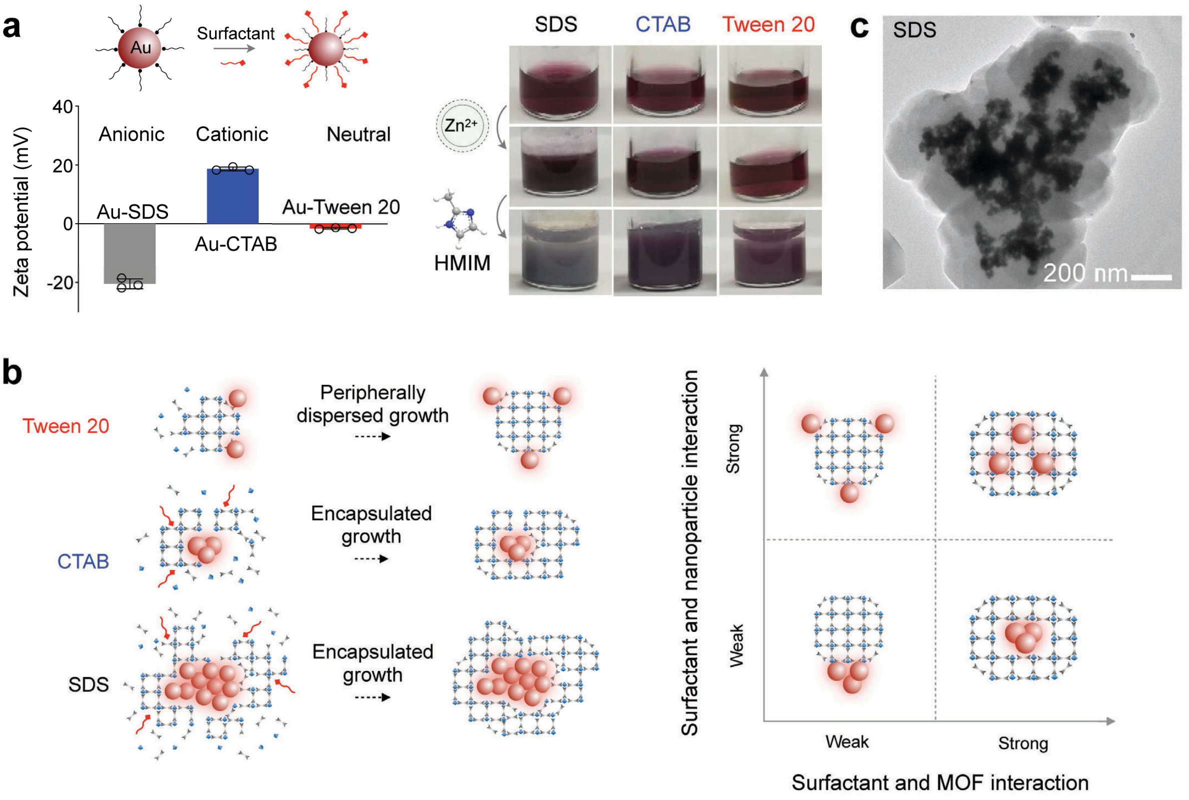

Supplementary Fig. 7: Surfactant-dependent nanoparticle integration.
a Left: zeta potential of Au nanospheres, coated with SDS, CTAB and Tween 20, respectively. Right: stepwise photographs of the reaction solutions, demonstrating the formation of STARs. **b** Nanoparticle integration into MOF hosts. Left: schematic processes for integrating different surfactant-coated nanoparticles into ZIF-8. Right: tunable nanoparticle distribution and organization in MOF hosts, as determined by the interactions of surfactants with MOF constituents and nanoparticles, respectively. **c** A typical TEM image of Au-ZIF-8 architecture, where the Au nanoparticles were coated with SDS. All measurements were performed in triplicate and the data are displayed as mean ± SD in **a**. TEM experiment was repeated three times independently with similar results in **c**. Source data are provided as a Source Data file.

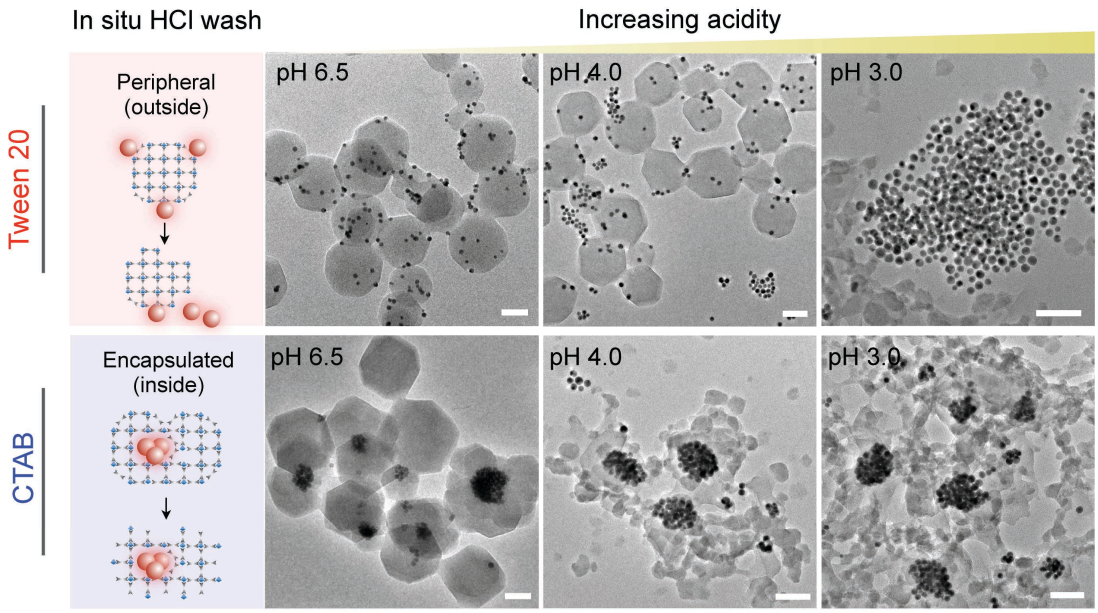

Supplementary Fig. 8: In situ acid washes of Au-ZIF-8 architectures.
With increasing buffer acidity, peripherally-associated nanoparticles (Tween 20-coated) were dislodged from the MOF host while the centrally-encapsulated nanoparticles (CTAB-coated) remained within the MOF host. Each TEM experiment was repeated three times independently with similar results. Scale bars, 50 nm.

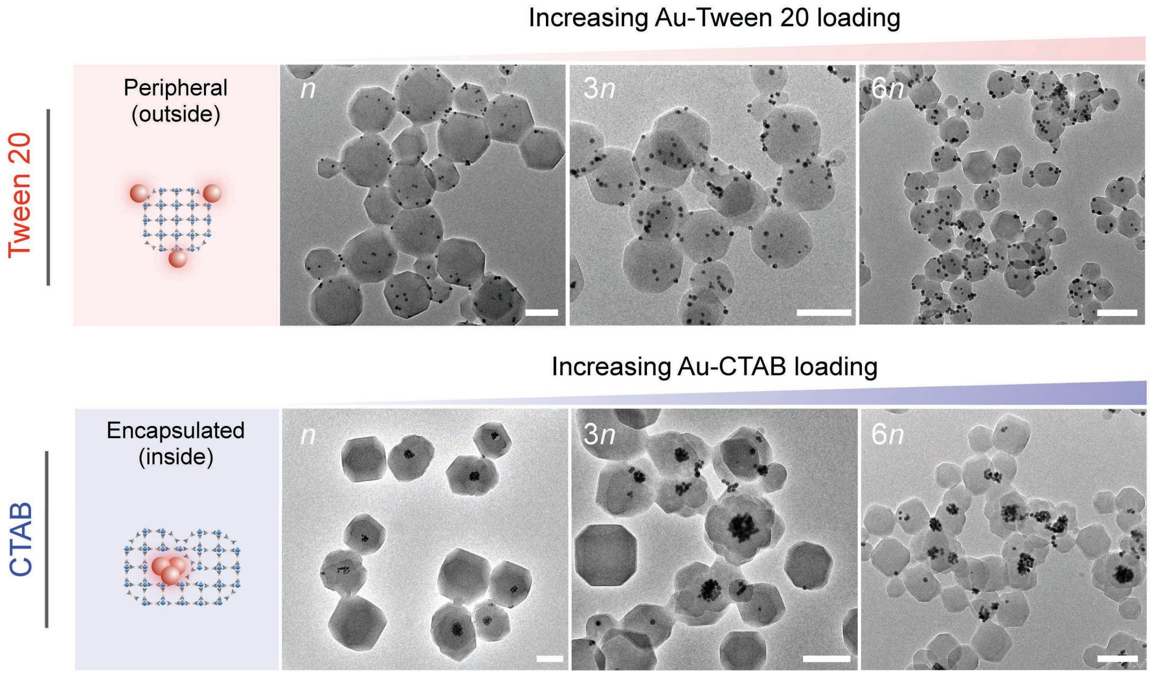

Supplementary Fig. 9: Au-ZIF-8 architectures prepared with varied Au loading.
Different-sized Au-ZIF-8 architectures were prepared by varying the Au loading (n = 5 µg). For Tween 20-coated Au, most of the Au nanospheres were peripherally associated with the MOF host. For CTAB-coated Au, the nanospheres were dominantly encapsulated within the MOF host. Each TEM experiment was repeated three times independently with similar results. Scale bars, 100 nm.

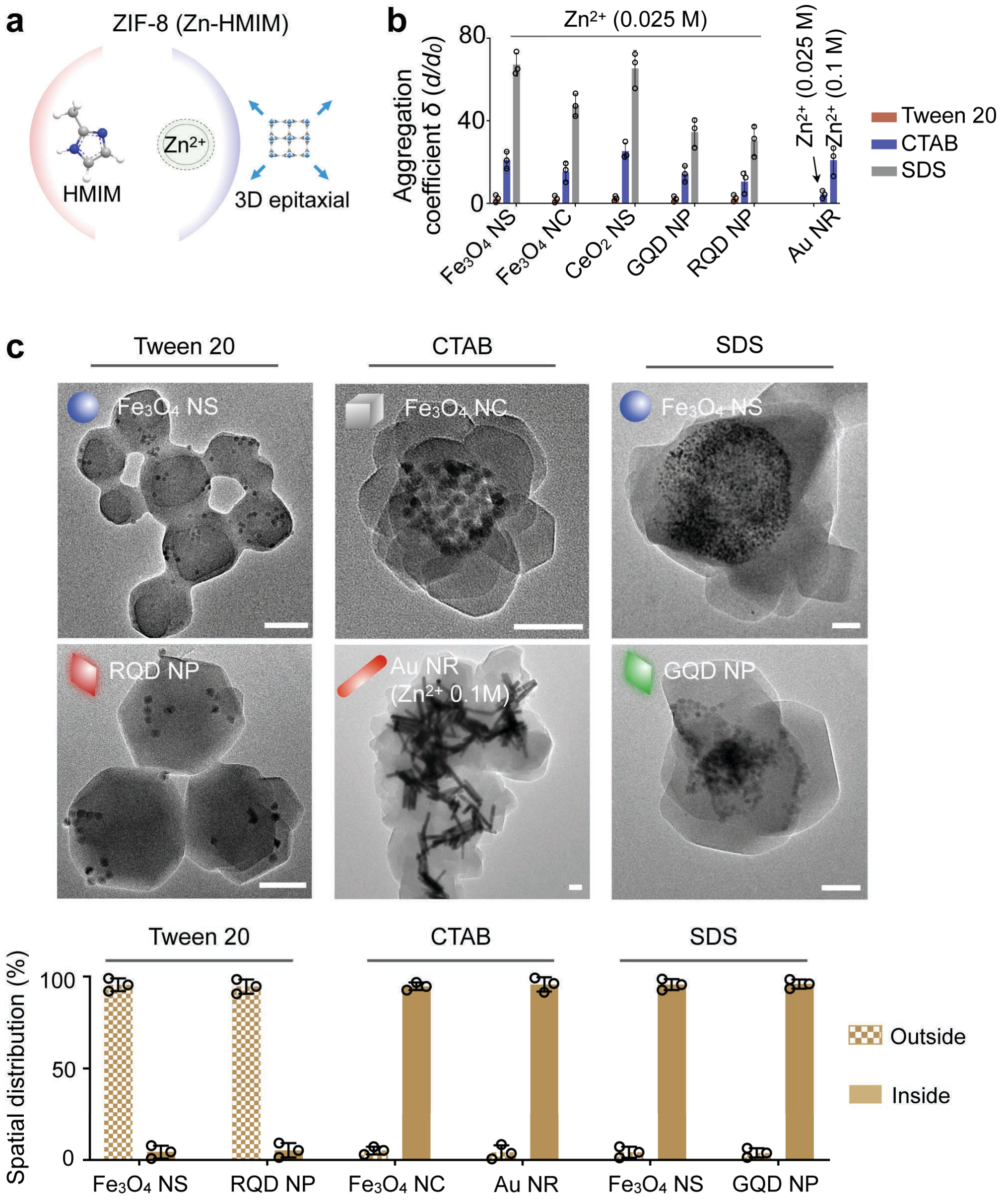

Supplementary Fig. 10: ZIF-8 based STARs.

a Metal node and organic linker for 3D epitaxial ZIF-8 formation. **b** Aggregation of surfactant-coated nanoparticles in the presence of Zn^{2+}. Nanoparticles were applied at 2 mg/ml. **c** TEM images of the STARs and corresponding analysis of nanoparticle spatial distribution through acid buffer wash (HCl buffer, pH = 4). The approach demonstrated good universality in tuning the spatial integration of diverse nanoparticles in ZIF-8. Each TEM experiment was repeated three times independently with similar results. NS, nanosphere. NC, nanocube. NP, nanopyramid. NR, nanorod. All measurements were performed in triplicate and the data are displayed as mean ± SD. Scale bars, 50 nm. Source data are provided as a Source Data file.

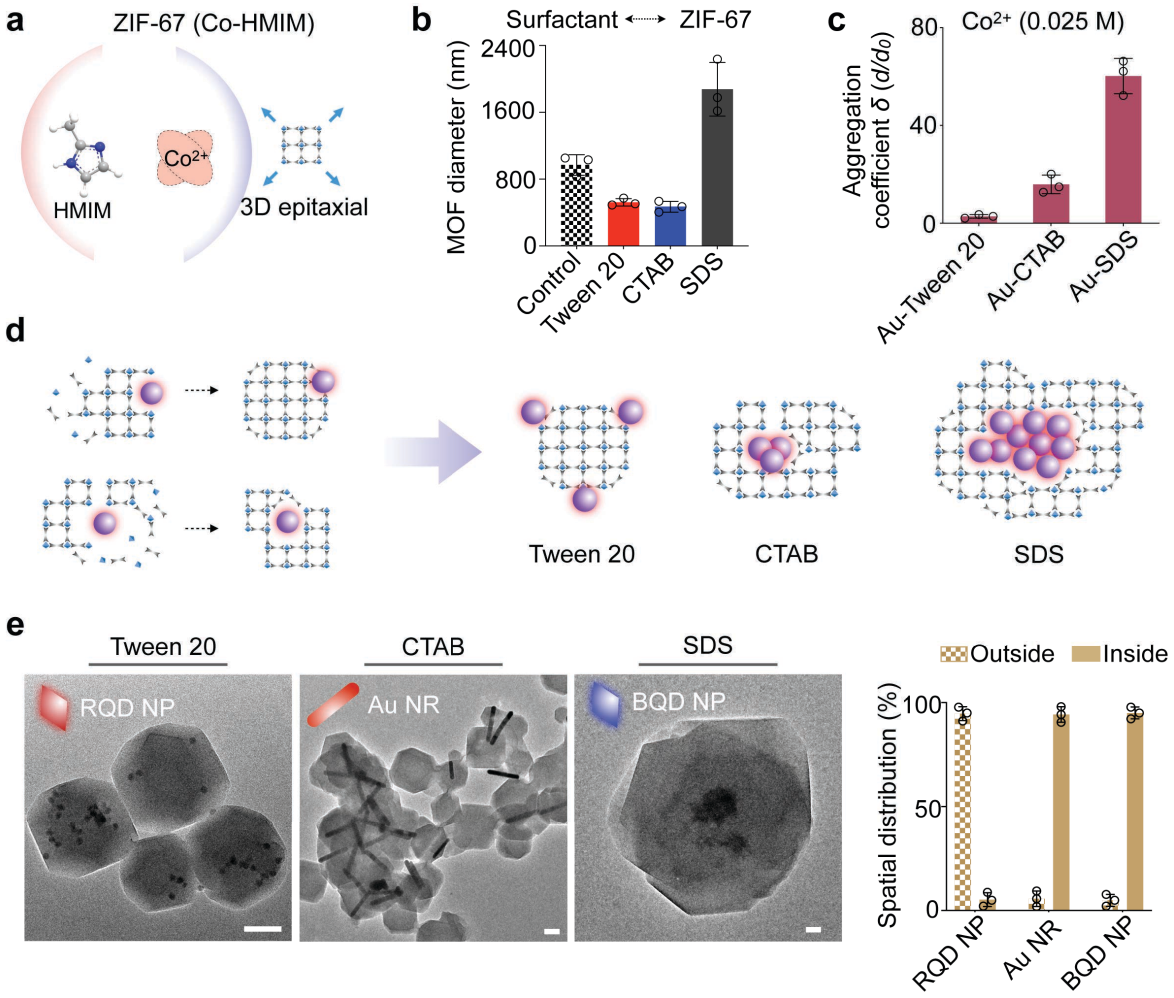

Supplementary Fig. 11: ZIF-67 based STARs.

a Metal node and organic linker for 3D epitaxial ZIF-67 formation. **b** Diameter of ZIF-67 particles in the presence of free surfactants (0.5 mM). A similar surfactant effect was observed on ZIF-67 growth to that on ZIF-8 growth (see Fig. 2c). **c** Aggregation of surfactant-coated Au nanospheres in the presence of Co^{2+}. Nanoparticles were applied at 2 mg/ml. **d** Schematic prediction of nanoparticle integration into ZIF-67. The nanoparticle distribution and organization could be predicated based on the surfactant interactions to nanoparticles and MOF, respectively. **e** TEM images of the STARs and corresponding analysis of nanoparticle spatial distribution through acid buffer wash (HCl buffer, pH = 4), consistent with the prediction. Each TEM experiment was repeated three times independently with similar results. NP, nanopyramid. NR, nanorod. All measurements were performed in triplicate and the data are displayed as mean ± SD. Scale bars, 50 nm. Source data are provided as a Source Data file.

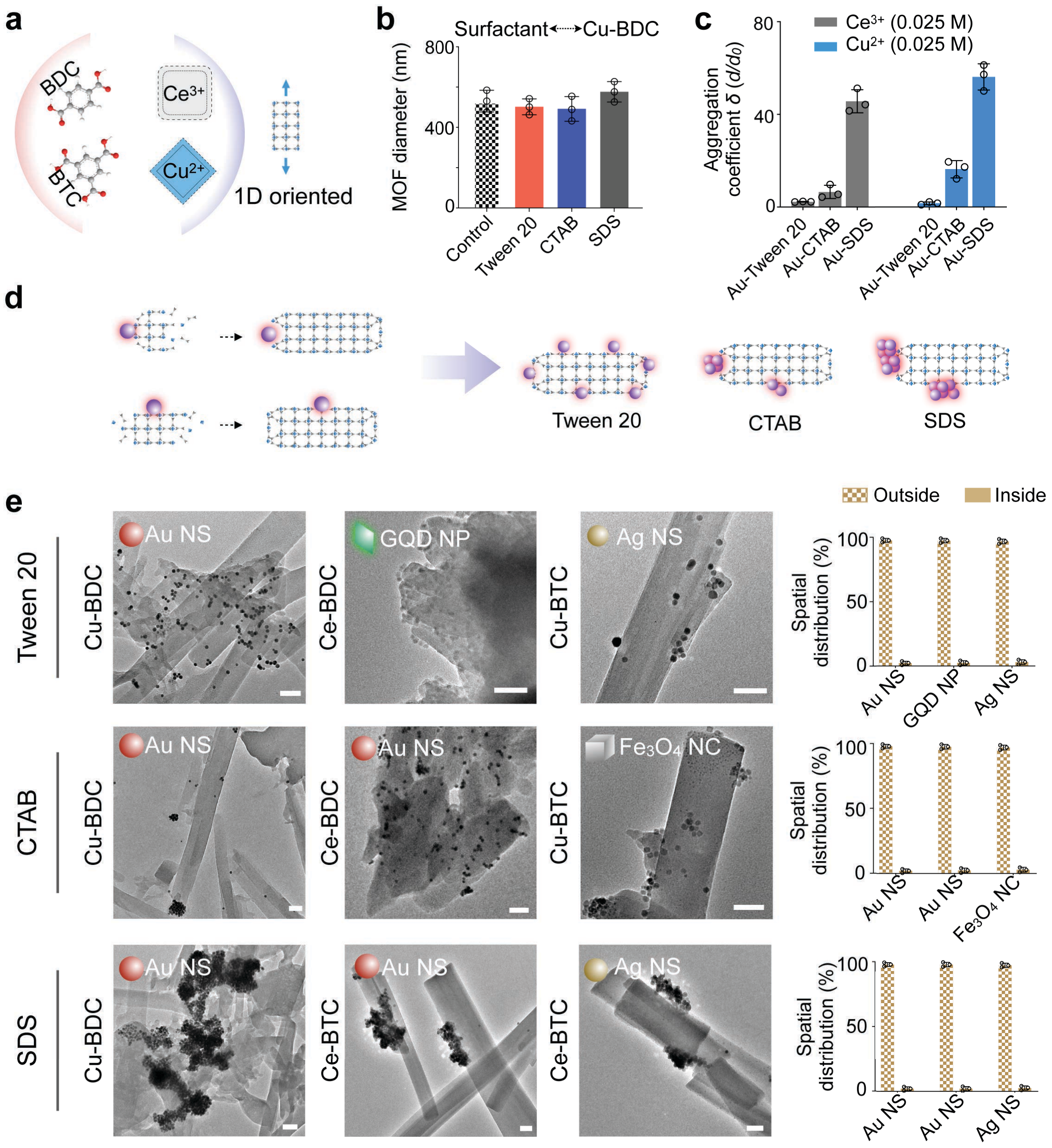

Supplementary Fig. 12: 1D-oriented STARs.

a Schematic of metal nodes and organic linkers for constructing 1D-oriented MOFs. **b** Surfactants showed weak interactions to the 1D-oriented MOFs, as verified by minimal changes to the MOF diameter in the presence of free surfactants (1 mM) using Cu-BDC as a model. **c** MOF constituents (e.g., Ce^{3+}, Cu^{2+}) induced nanoparticle aggregation. Nanoparticle aggregation was surfactant-dependent (aggregation coefficient: SDS > CTAB > Tween 20). **d** Schematic prediction of nanoparticle integration in 1D-oriented MOFs. As predicated, the weak interaction of surfactants to 1D-oriented MOFs induces peripheral nanoparticle association with the MOF hosts. Nanoparticle aggregation is surfactant-dependent. **e** TEM images of the STARs and corresponding analysis of nanoparticle spatial distribution through buffer wash. The nanoparticles were dominantly outside the MOF hosts, dispersed (Tween 20) or aggregated (CTAB, SDS), which agreed well with the prediction. HCl buffer (pH = 3) and NaOH buffer (pH = 9) were used for the analysis of BTC-based and BDC-based STARs, respectively. Each TEM experiment was repeated three times independently with similar results. NS, nanosphere. NP, nanopyramid. NC, nanocube. All measurements were performed in triplicate and the data are displayed as mean ± SD. Scale bars, 50 nm. Source data are provided as a Source Data file.

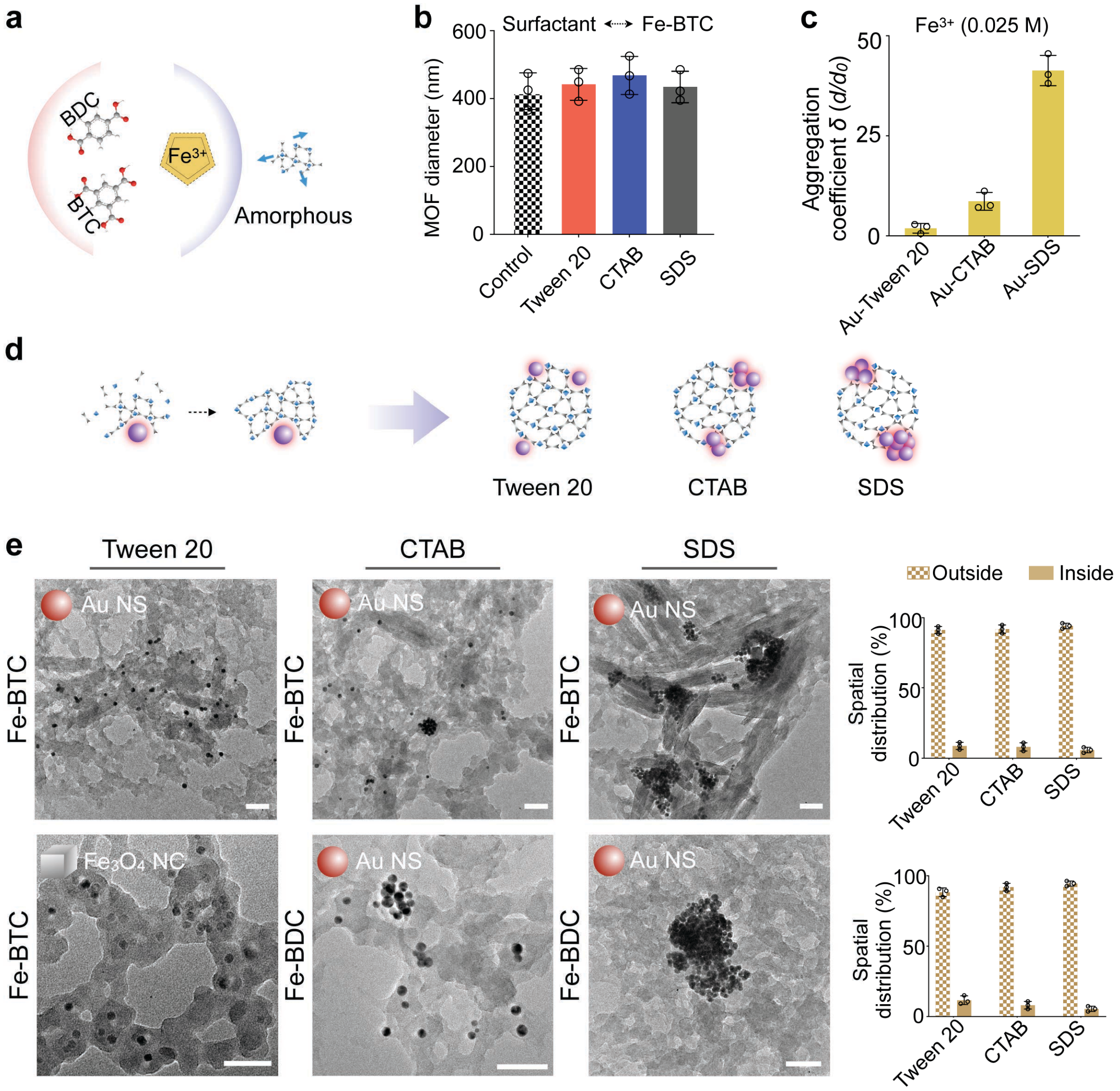

Supplementary Fig. 13: Amorphous STARs.

a Schematic of metal nodes and organic linkers for constructing amorphous Fe-BDC and Fe-BTC. **b** Surfactants showed weak interactions to the hosts, as verified by minimal changes to the formed diameter in the presence of free surfactants (1 mM) using Fe-BTC as a model. **c** Constituents (e.g., Fe^{3+}) induced nanoparticle aggregation. Nanoparticle aggregation was surfactant-dependent. **d** Schematic prediction of nanoparticle integration in amorphous products. As predicated, the weak interaction of surfactants to hosts induces peripheral nanoparticle association. Nanoparticle aggregation is surfactant-dependent. **e** TEM images of the STARs and corresponding analysis of nanoparticle spatial distribution through buffer wash. Most nanoparticles are found outside the hosts, dispersed (Tween 20) or aggregated (CTAB, SDS), which agreed with the prediction. HCl buffer (pH = 2) and NaOH buffer (pH = 9.5) were used for the analysis of BTC-based and BDC-based STARs, respectively. Each TEM experiment was repeated three times independently with similar results. NS, nanosphere. NC, nanocube. All measurements were performed in triplicate and the data are displayed as mean ± SD. Scale bars, 50 nm. Source data are provided as a Source Data file.

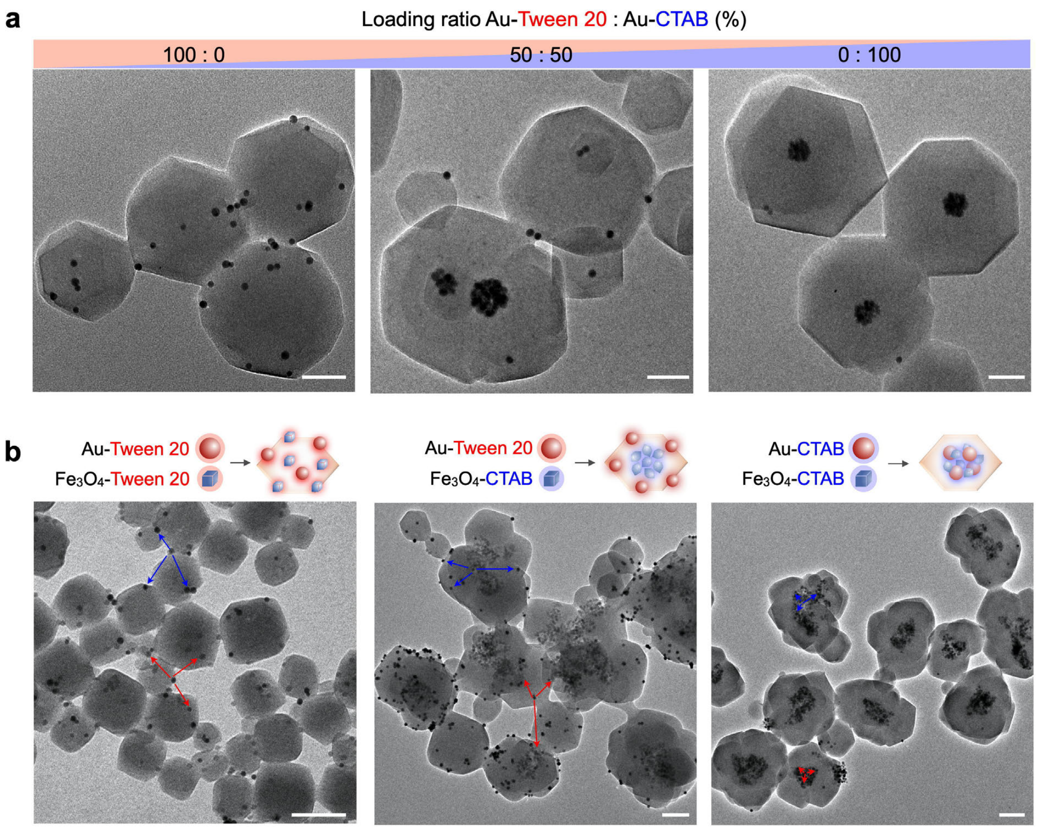

Supplementary Fig. 14: Nanoparticle spatial distribution and organization in MOFs.
a Single-nanoparticle system. Au-ZIF-8 assemblies were prepared by regulating the loading ratio of Au-Tween 20 and Au-CTAB. The prepared assemblies showed controlled nanoparticle distribution and organization. Specifically, when more Tween 20-coated nanoparticles were loaded, more nanoparticles resided as mono-dispersed outside the MOFs; when more CTAB-coated nanoparticles were used, more nanoparticle aggregates were observed encapsulated within the MOFs. Scale bars, 50 nm. **b** Multi-nanoparticle system. By surfactant matching, Au nanospheres and Fe_3O_4 nanocubes were variedly integrated into the MOF system. TEM analysis verified the nanoparticle spatial distribution and organization within the MOF structure. The nanoparticle aggregation status was characterized by measuring the distance between nanoparticles (inter-particle distance). For instance, the three red arrows mark the distances from one Au nanosphere to three neighboring Fe_3O_4 nanocubes and the three blue arrows mark the distances from one Fe_3O_4 nanocube to three neighboring Au nanospheres. Each TEM experiment was repeated three times independently with similar results. Scale bars, 100 nm.

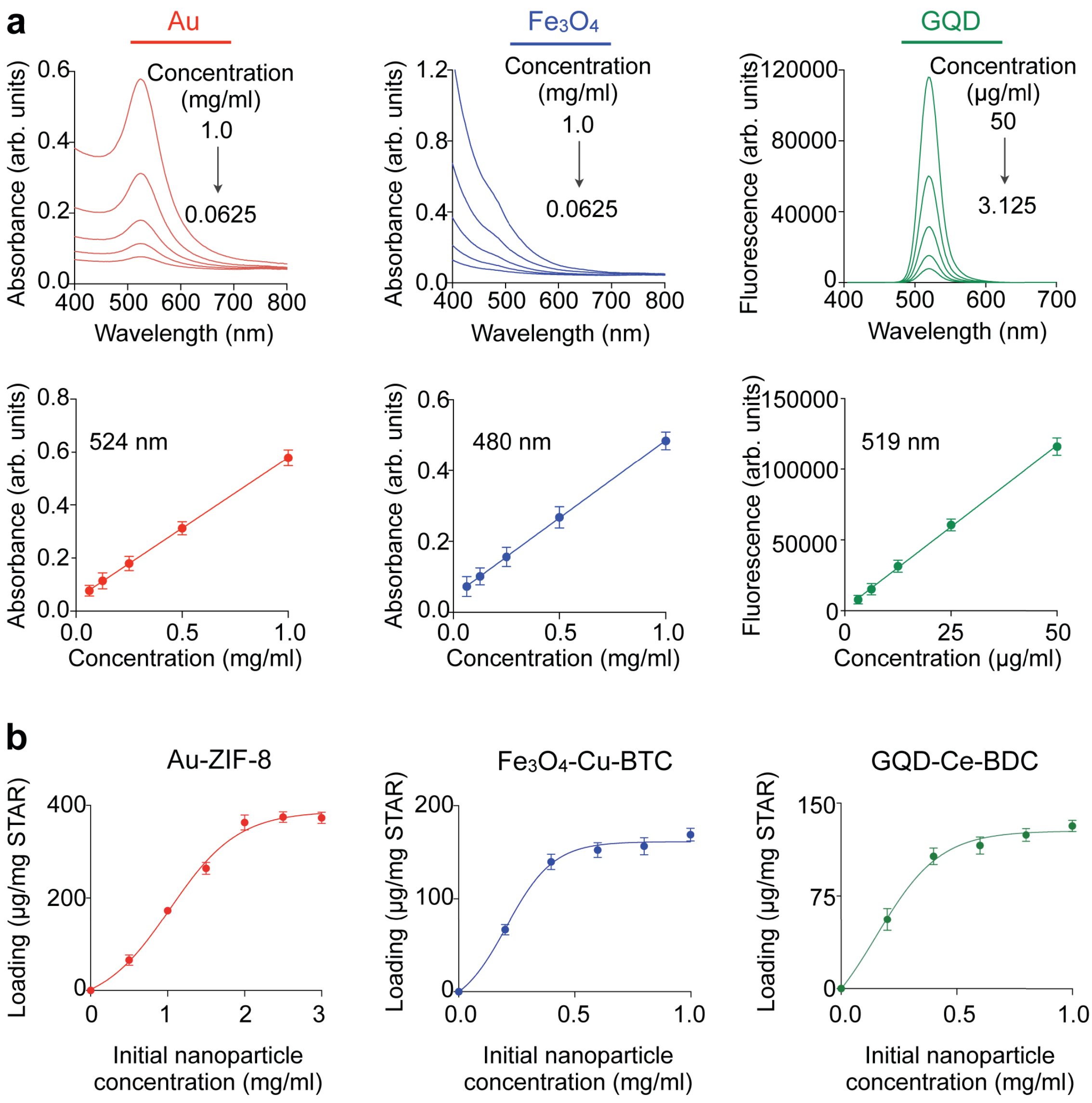

Supplementary Fig. 15: Nanoparticle integration efficiency.

a Standard curves were measured to determine nanoparticle concentrations: UV-Vis absorbance at 524 nm for Au nanosphere, UV-Vis absorbance at 480 nm for Fe_3O_4 nanocube, and fluorescence at 519 nm for GQD. Standard curves were obtained through linear regression with 95% confidence interval. **b** Controlled nanoparticle loading into STARs. By increasing the initial nanoparticle concentration in the reaction solution, more nanoparticles were loaded into STARs. All measurements were performed in triplicate and the data are displayed as mean ± SD. arb. units, arbitrary units. Source data are provided as a Source Data file.

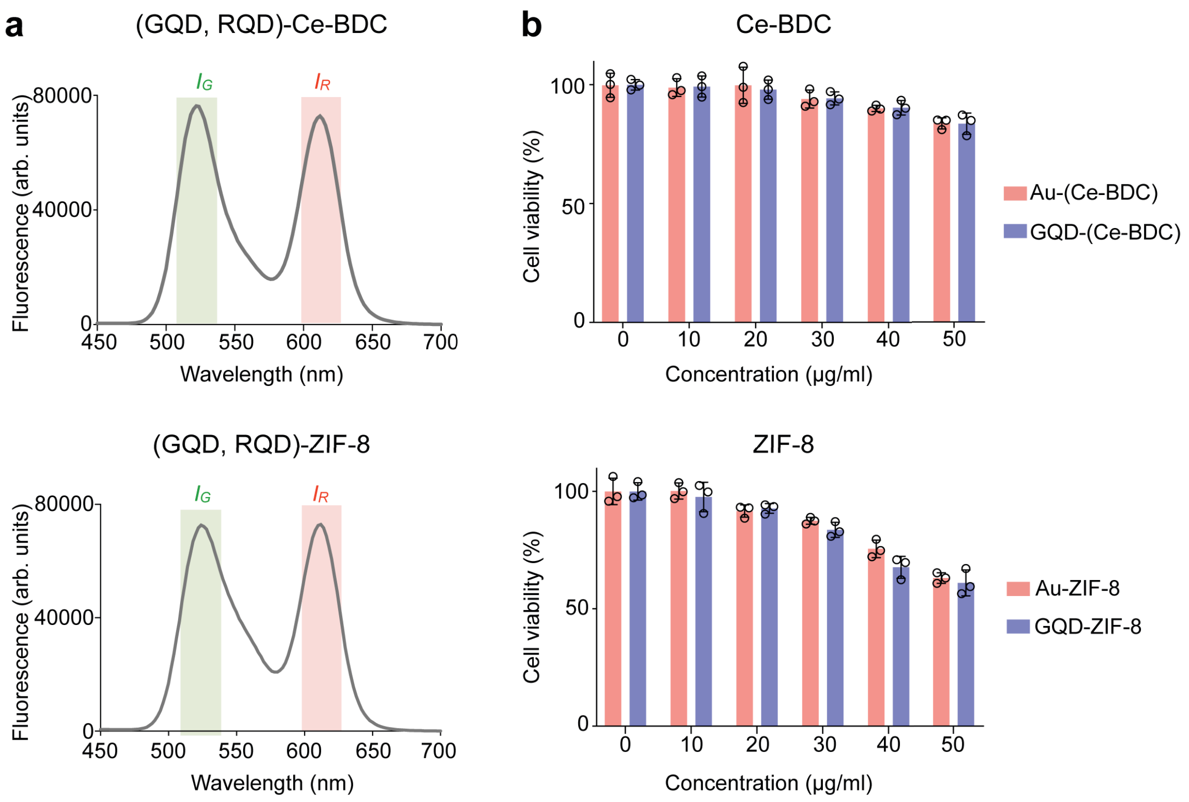

Supplementary Fig. 16: Effects of MOF morphology.

a Fluorescence spectra of STAR composites. QDs were incorporated into 1D-oriented Ce-BDC (top) or 3D epitaxial ZIF-8 (bottom). Both products were quantified to contain an equal amount of respective QDs. The products showed a similar fluorescence profile, indicating the negligible influence of MOF host morphology on the fluorescence properties of the STAR composites. **b** Cellular toxicity of STAR composites. Different STAR composites were incubated with epithelial cells (A431) for 24 hours. Cellular toxicity was evaluated through the MTS proliferation assay. The choice of MOF hosts (more so than the choice of nanoparticles) exerted a strong effect on cellular toxicity. All measurements were performed in triplicate and the data are displayed as mean ± SD. arb. units, arbitrary units. Source data are provided as a Source Data file.

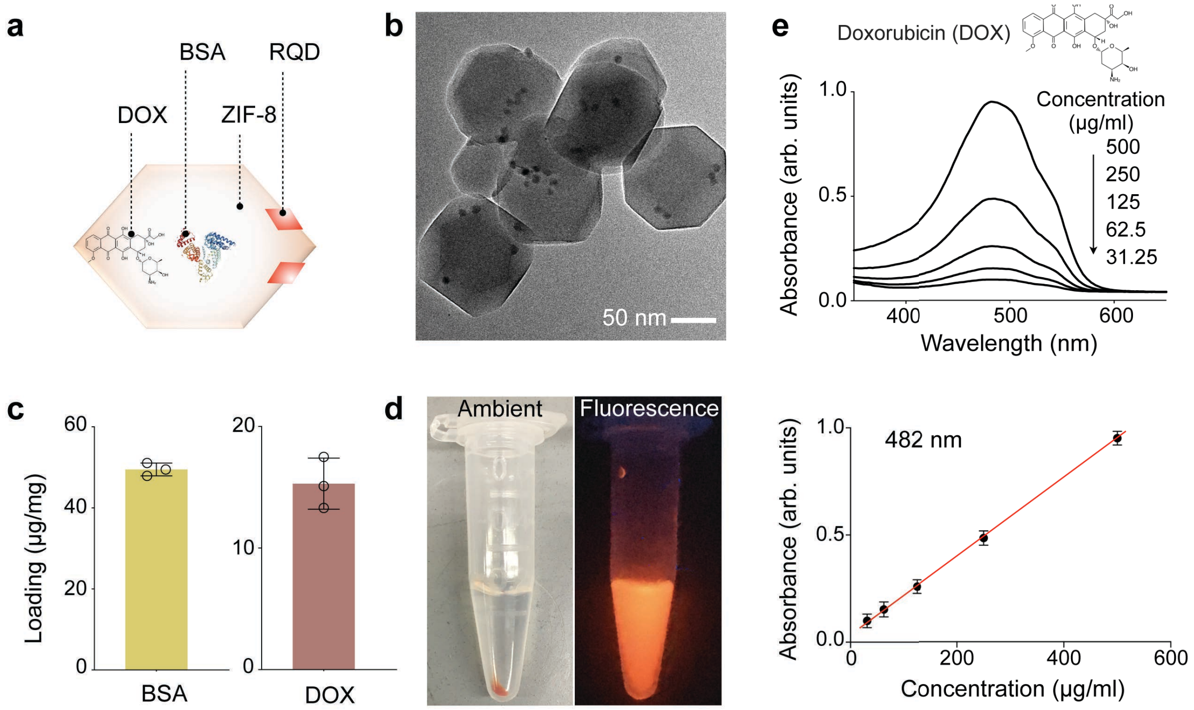

Supplementary Fig. 17: STAR incorporation of biomolecules.
a Schematic of (RQD-Tween 20)-ZIF-8 with co-integrated therapeutic drug DOX and protein BSA. **b** Typical TEM image of the composite. TEM experiment was repeated three times independently with similar results. **c** Loading amount of BSA and DOX. **d** Photographs of the developed composite in water. The composite is red under ambient light due to the loaded DOX and demonstrates fluorescence properties, consistent with its RQD constituent. **e** UV-Vis absorption spectra of DOX and the corresponding standard curve (measured at 482 nm) for DOX quantification. Standard curves were obtained through linear regression with 95% confidence interval. All measurements were performed in triplicate and the data are displayed as mean ± SD. arb. units, arbitrary units. Source data are provided as a Source Data file.

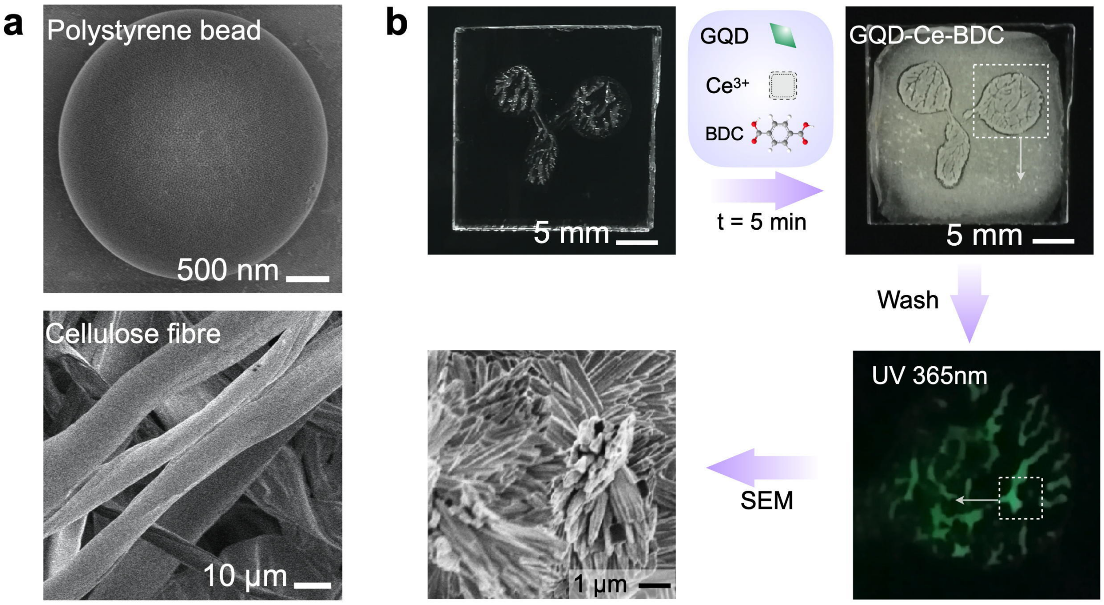

Supplementary Fig. 18: STAR growth on different substrates.

a Scanning electron microscopy (SEM) images of pristine polystyrene bead and cellulose fiber. **b** Templated growth of GQD-Ce-BDC along a seeding pattern made of triglycerides on a glass slide. Each experiment was repeated three times independently with similar results.

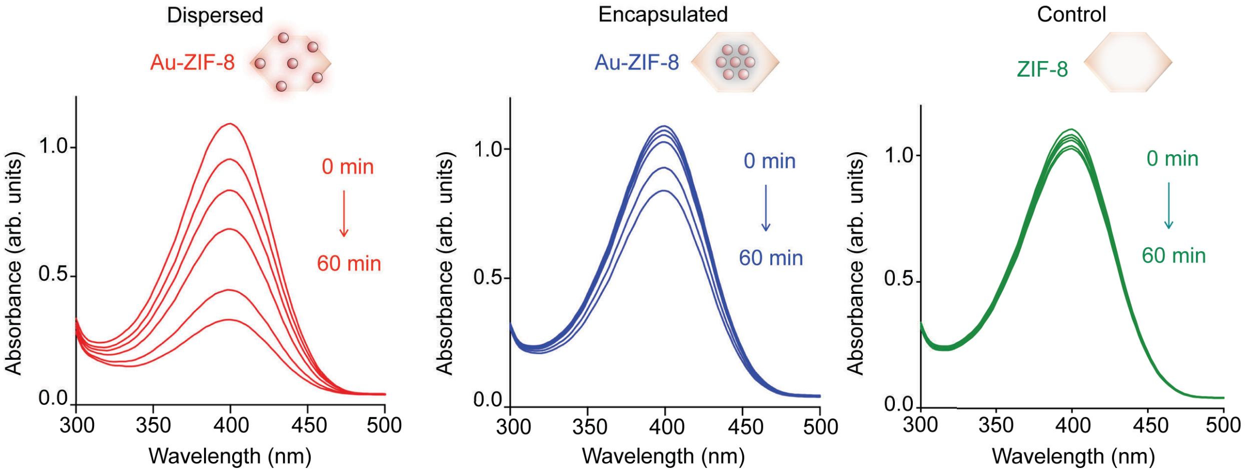

Supplementary Fig. 19: STAR as a nanocatalyst.

Two types of Au-ZIF-8 with different spatial distribution of Au nanospheres, dispersed vs. encapsulated, were prepared as catalysts for the transformation of 4-nitrophenol (4-NP) to 4-aminophenol (4-AP) in the presence of reductive $NaBH_4$. The dispersed version was prepared through Au-Tween 20 and the encapsulated through Au-CTAB. By absorbance measurement, we demonstrated that the dispersed STAR has a higher catalytic efficiency than the encapsulated version. The pure ZIF-8 control showed negligible catalytic activity. arb. units, arbitrary units. Source data are provided as a Source Data file.

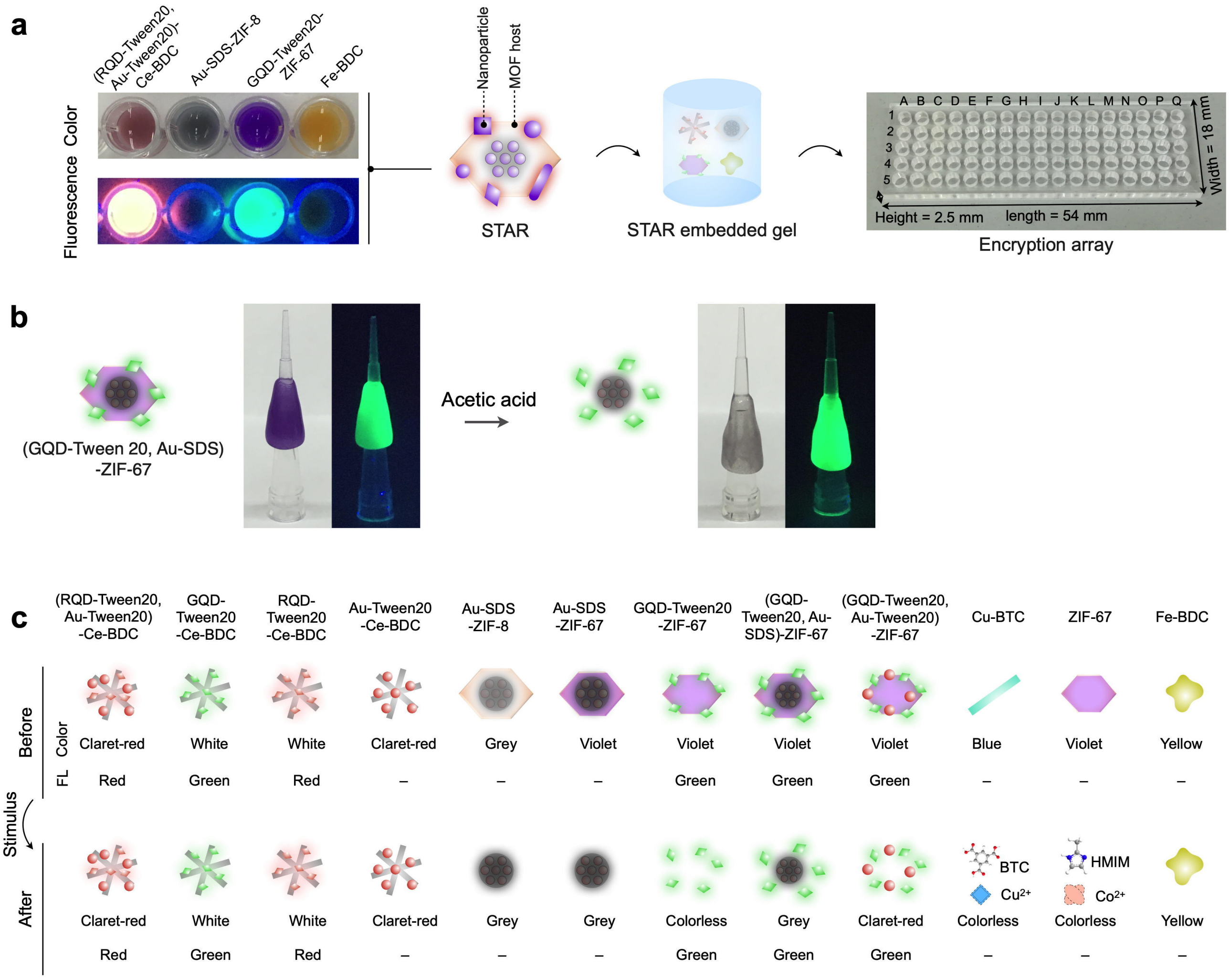

Supplementary Fig. 20: Encryption array.

a Typical STARs with unique optical features (color and fluorescence) for the development of the encryption chip. STARs are embedded within a polyacrylamide gel and patterned on a PMMA array. **b** Stimulus-induced response. (GQD-Tween 20, Au-SDS)-ZIF-67 was used. It appeared dark violet under ambient light and showed green fluorescence under UV excitation. Upon stimulus treatment (2% acetic acid), ZIF-67 was destroyed and turned colorless. Consequently, the aggregated Au-SDS became visible and appeared grey under ambient light. The structure continued to emit green fluorescence as the GQD remained intact after the stimulus treatment. **c** Different STARs used for information encryption. The assemblies were made of different combinations of nanoparticles and MOFs, and demonstrated different optical responses (color and fluorescence) upon stimulus.

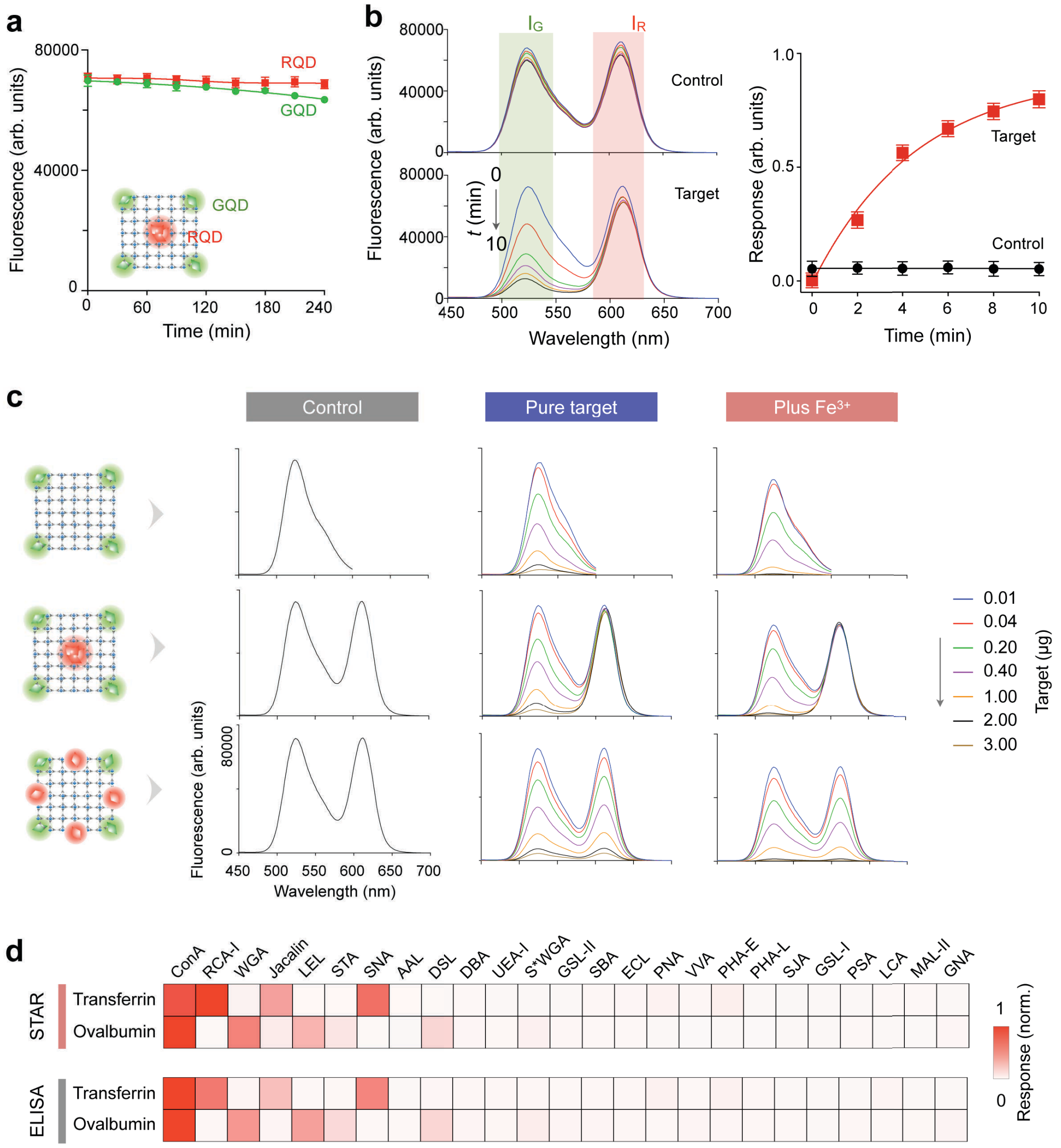

Supplementary Fig. 21: STAR for enhanced detection of glycans.

a Minimal leaching of QD probes in STAR. To evaluate the extent of nanoparticle leaching from MOF, we prepared a dual-probe STAR (RQD inside and GQD outside). We incubated the structure in the assay buffer, and periodically extracted the composite to monitor its fluorescence intensities over time. **b** Time-resolved fluorescence spectra of the dual-probe STAR in the absence (control) and presence of a model target (transferrin). The response was determined by $R = 1 - (S_t / S_o)$ and $S = I_G / I_R$, where S_0 is the initial ratiometric fluorescence and S_t is the ratiometric fluorescence at time t. I_G and I_R are the green and red fluorescence intensities, respectively. All measurements were performed in triplicate and the data are displayed as mean ± SD. **c** The performance of three types of STARs. Different STARs were prepared with quantum dots (i.e., QD-ZIF-8) and applied to analyze the target in the absence (pure) and presence of an interfering and quenching agent (Fe^{3+}, 1 μM). **d** Glycan profiling using 25 lectins on two model glycoproteins, transferrin and ovalbumin. Measurements were performed through both STAR and ELISA assay. All measurements were made with an equal concentration of protein component, and performed relative to sample-matched no-lectin controls. The datasets are normalized individually to the highest signal for each analyte and the mean values are presented as heatmaps. arb. units, arbitrary units. Source data are provided as a Source Data file.

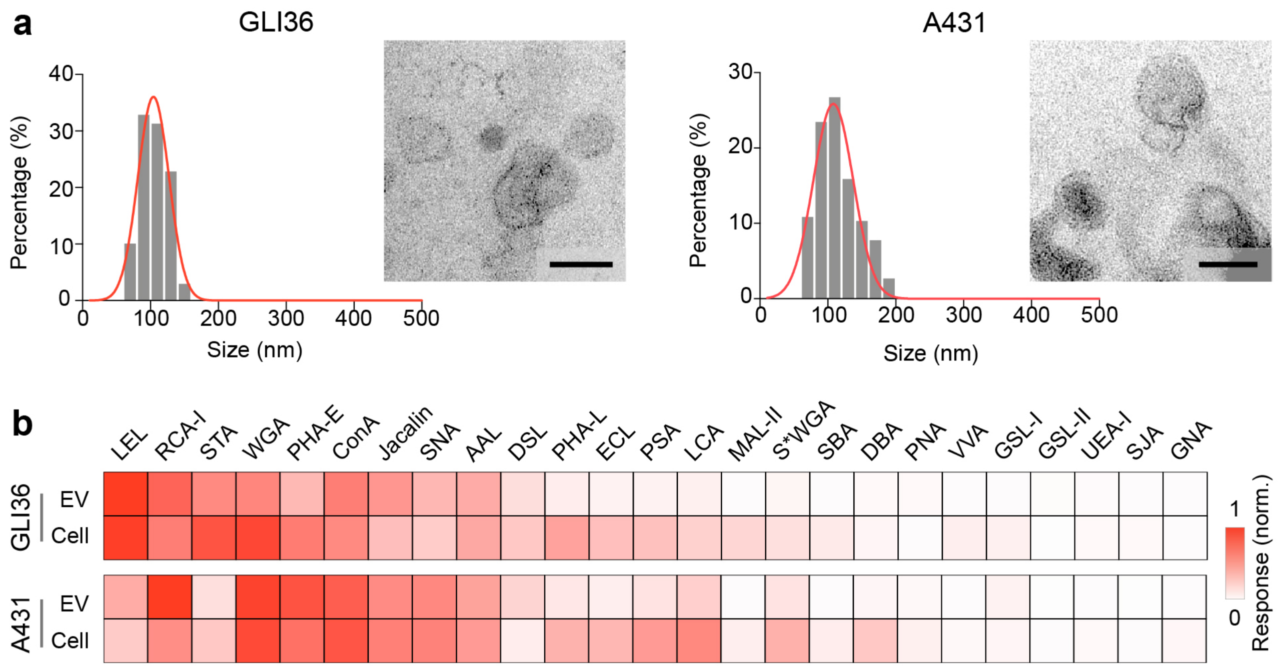

Supplementary Fig. 22: EV glycan profiling using the STAR assay.
a Extracellular vesicles isolated from brain glial cells (GLI36) and skin epithelial cells (A431) culture medium. All EVs were characterized with nanoparticle tracking analysis and TEM. Each experiment was repeated three times independently with similar results. Scale bars, 100 nm. **b** Multiplexed glycan profiling using 25 lectins on vesicles derived from brain glial cells (GLI36) and skin epithelial cells (A431). Measurements were performed with an equal vesicle concentration (5 × 10^8/ml) across samples through the multiplexed STAR platform. Corresponding cell surface glycans were profiled through ELISA. The datasets are normalized individually to the highest signal for each sample and the mean values are presented as heatmaps. Source data are provided as a Source Data file.

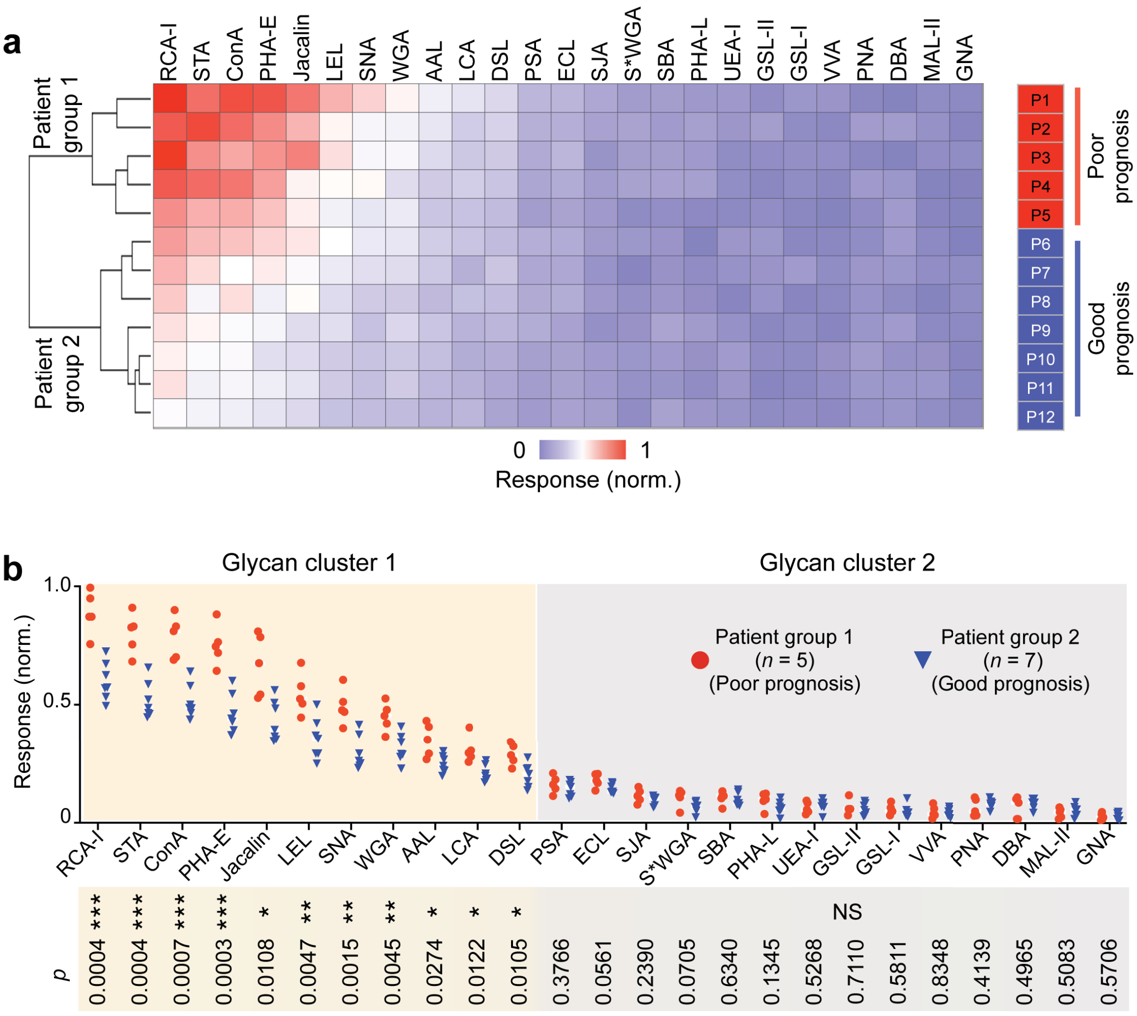

Supplementary Fig. 23: Clinical analysis of patient ascites.

a Clinical glycan profiling using 25 lectins on ascites samples of colorectal cancer cohort (*n* = 12 patients). Measurements were performed through the STAR assay. All measurements were made with an equal volume of ascites (5 µl) across samples, and performed relative to sample-matched no-lectin controls. The datasets were normalized globally to the highest signal. Hierarchical clustering analysis of the patient specimens categorized the samples into two distinct groups, showing a good concordance with the independent clinical evaluation of patient prognosis (poor and good prognosis). **b** Lectin responses of poor and good prognosis patient groups were compared by two-tailed Student's *t*-test. *p*-values are indicated and $p < 0.05$ is determined as significant. (NS, not significant; *$p < 0.05$, **$p < 0.01$, ***$p < 0.001$). Source data are provided as a Source Data file.

Supplementary Table 1: STAR composition and arrangement in the encryption device.

	1	2	3	4	5
A	(RQD-Tween20, Au-Tween20)-Ce-BDC	1.(RQD-Tween20, Au-Tween20)-Ce-BDC (95%) 2.Cu-BTC (5%)	1.(RQD-Tween20, Au-Tween20)-Ce-BDC (95%) 2.Cu-BTC (5%)	1.(RQD-Tween20, Au-Tween20)-Ce-BDC (95%) 2.Cu-BTC (5%)	1.(RQD-Tween20, Au-Tween20)-Ce-BDC (95%) 2.Cu-BTC (5%)
B	GQD-Tween20-ZIF-67	ZIF-67	GQD-Tween20-ZIF-67	ZIF-67	GQD-Tween20-ZIF-67
C	RQD-Tween20-Ce-BDC	RQD-Tween20-Ce-BDC	RQD-Tween20-Ce-BDC	RQD-Tween20-Ce-BDC	RQD-Tween20-Ce-BDC
D	Au-SDS-ZIF-8	Au-SDS-ZIF-8	Au-SDS-ZIF-8	Au-SDS-ZIF-8	Au-SDS-ZIF-8
E	GQD-Tween20-ZIF-67	GQD-Tween20-ZIF-67	GQD-Tween20-ZIF-67	GQD-Tween20-ZIF-67	GQD-Tween20-ZIF-67
F	Fe-BDC	Fe-BDC	Fe-BDC	Fe-BDC	Fe-BDC
G	1. Cu-BTC(70%) 2. GQD-Tween20-Ce-BDC(30%)	1. Cu-BTC(70%) 2. GQD-Tween20-Ce-BDC(30%)	1. Cu-BTC(70%) 2. GQD-Tween20-Ce-BDC(30%)	1. Cu-BTC(70%) 2. GQD-Tween20-Ce-BDC(30%)	1. Cu-BTC(70%) 2. GQD-Tween20-Ce-BDC(30%)
H	1. Cu-BTC(60%) 2. GQD-Tween20-Ce-BDC(40%)	Cu-BTC	Cu-BTC	Cu-BTC	1. Cu-BTC(60%) 2. GQD-Tween20-Ce-BDC(40%)
I	RQD-Tween20-Ce-BDC	RQD-Tween20-Ce-BDC	RQD-Tween20-Ce-BDC	RQD-Tween20-Ce-BDC	RQD-Tween20-Ce-BDC
J	Au-Tween20-Ce-BDC	Au-Tween20-Ce-BDC	Au-Tween20-Ce-BDC	Au-Tween20-Ce-BDC	Au-Tween20-Ce-BDC
K	(GQD-Tween20, Au-Tween20)-ZIF-67	(GQD-Tween20, Au-Tween20)-ZIF-67	(GQD-Tween20, Au-Tween20)-ZIF-67	(GQD-Tween20, Au-Tween20)-ZIF-67	(GQD-Tween20, Au-Tween20)-ZIF-67
L	1. (GQD-Tween20, Au-Tween20)-ZIF-67 2. Cu-BTC(5%)	ZIF-67	1. (GQD-Tween20, Au-Tween20)-ZIF-67 2. Cu-BTC(5%)	ZIF-67	(GQD-Tween20, Au-Tween20)-ZIF-67
M	1. Fe-BDC (50%) 2. RQD-Tween20-Ce-BDC(45%) 3.Cu-BTC(5%)	Fe-BDC	1. Fe-BDC (50%) 2. RQD-Tween20-Ce-BDC(45%) 3.Cu-BTC(5%)	1. Fe-BDC (50%) 2. RQD-Tween20-Ce-BDC(45%) 3.Cu-BTC(5%)	1. Fe-BDC (50%) 2. RQD-Tween20-Ce-BDC(50%)
N	Cu-BTC	Cu-BTC	Cu-BTC	Cu-BTC	Cu-BTC
O	1. Fe-BDC (50%) 2. RQD-Tween20-Ce-BDC(50%)	1. Fe-BDC (50%) 2. RQD-Tween20-Ce-BDC(45%) 3.Cu-BTC(5%)	1. Fe-BDC (50%) 2. RQD-Tween20-Ce-BDC(50%)	Fe-BDC	1. Fe-BDC (50%) 2. RQD-Tween20-Ce-BDC(50%)
P	(GQD-Tween20, Au-SDS)-ZIF-67	Au-SDS-ZIF-67	(GQD-Tween20, Au-SDS)-ZIF-67	Au-SDS-ZIF-67	(GQD-Tween20, Au-SDS)-ZIF-67
Q	(RQD-Tween20, Au-Tween20)-Ce-BDC	(RQD-Tween20, Au-Tween20)-Ce-BDC	(RQD-Tween20, Au-Tween20)-Ce-BDC	(RQD-Tween20, Au-Tween20)-Ce-BDC	(RQD-Tween20, Au-Tween20)-Ce-BDC

STAR loading in each well is 0.5 mg. Number in parenthesis denotes weight percentage.

Supplementary Table 2: List of lectins and their targeted glycan specificities.

Abbreviation	Source/Name	Preferred glycan specificity
ConA	*Concanavalin A*	αMan, αGlc
SBA	*Glycine max (soybean) agglutinin*	α>βGalNAc
WGA	*Triticum vulgaris (wheat germ) agglutinin*	GlcNAc, SA
DBA	*Dolichos biflorus agglutinin*	αGalNAc
UEA-I	*Ulex europaeus agglutinin I*	(α-1,2) Fuc
RCA120	*Ricinus communis agglutinin*	Gal
PNA	*Arachis hypogaea (peanut) agglutinin*	Galβ3GalNAc
GSL-I	*Griffonia (Bandeiraea) simplicifolia lectin I*	αGal, αGalNAc
PSA	*Pisum sativum agglutinin*	αMan, αGlc
LCA	*Lens culinaris agglutinin*	αMan, αGlc
PHA-E	*Phaseolus vulgaris Erythroagglutinin*	Galβ4GlcNAcβ2Manα6(GlcNAcβ4)<br>(GlcNAcβ4Manα3)Manβ4
PHA-L	*Phaseolus vulgaris Leucoagglutinin*	Galβ4GlcNAcβ6(GlcNAcβ2Manα3)Manα3
SJA	*Sophora japonica (Japanese Pagoda Tree)*	βGalNAc
SWGA	*Wheat germ agglutinin, succinylated*	GlcNAc
GSL-II	*Griffonia (Bandeiraea) simplicifolia lectin II*	α or βGlcNAc
DSL	*Datura Stramonium lectin*	(GlcNAc)2-4
ECL	*Erythrina cristagalli lectin*	Galβ4GlcNAc
Jacalin	*Artocarpus integrifolia (Jackfruit)*	Galβ3GalNAc
LEL	*Lycopersicon esculentum (tomato) lectin*	(GlcNAc) 2-4
STA	*Solanum tuberosum (potatoe) lectin*	(GlcNAc) 2-4
VVA	*Vicia villosa agglutinin*	GalNAc
SNA	*Sambucus Nigra Lectin*	(α-2,6) SA
MAL-II	*Maackia Amurensis Lectin II*	(α-2,3) SA
AAL	*Aleuria aurantia lectin*	(α-1,3) or (α-1,6) Fuc
GNA	*Galanthus nivalis agglutinin*	(α-1,3) Man

Glycan abbreviations:

Fuc	L-Fucose	Man	Mannose	GalNAc	N-Acetylgalactosamine
Gal	D-Galactose	SA	Sialic Acid	GlcNAc	N-Acetylglucosamine
Glc	D-Glucose				

SUPPLEMENTARY REFERENCES

1. Corrin, M. L. & Harkins, W. D. The effect of salts on the critical concentration for the formation of micelles in colloidal electrolytes[1]. *J. Am. Chem. Soc.* **69**, 683-688 (1947).
2. Qazi, M. J. et al. Influence of surfactants on sodium chloride crystallization in confinement. *Langmuir* **33**, 4260-4268 (2017).
