# Supplementary material for: Surfactant-guided spatial assembly of nano-architectures for molecular profiling of extracellular vesicles
Source: Nat Commun. 2021 Jun 30;12:4039. doi: 10.1038/s41467-021-23759-9 (PMC8245598; doi:10.1038/s41467-021-23759-9)
Supplement: Supplementary file 2 — Reporting Summary [file 41467_2021_23759_MOESM2_ESM.pdf]

nature research

Corresponding author(s): Huilin Shao

Last updated by author(s): Apr 29, 2021

Reporting Summary

Nature Research wishes to improve the reproducibility of the work that we publish. This form provides structure for consistency and transparency in reporting. For further information on Nature Research policies, see our Editorial Policies and the Editorial Policy Checklist.

Statistics

For all statistical analyses, confirm that the following items are present in the figure legend, table legend, main text, or Methods section.

n/a	Confirmed	
☐	☒	The exact sample size (n) for each experimental group/condition, given as a discrete number and unit of measurement
☐	☒	A statement on whether measurements were taken from distinct samples or whether the same sample was measured repeatedly
☐	☒	The statistical test(s) used AND whether they are one- or two-sided<br>*Only common tests should be described solely by name; describe more complex techniques in the Methods section.*
☐	☒	A description of all covariates tested
☐	☒	A description of any assumptions or corrections, such as tests of normality and adjustment for multiple comparisons
☐	☒	A full description of the statistical parameters including central tendency (e.g. means) or other basic estimates (e.g. regression coefficient) AND variation (e.g. standard deviation) or associated estimates of uncertainty (e.g. confidence intervals)
☐	☒	For null hypothesis testing, the test statistic (e.g. F, t, r) with confidence intervals, effect sizes, degrees of freedom and P value noted<br>*Give P values as exact values whenever suitable.*
☒	☐	For Bayesian analysis, information on the choice of priors and Markov chain Monte Carlo settings
☒	☐	For hierarchical and complex designs, identification of the appropriate level for tests and full reporting of outcomes
☐	☒	Estimates of effect sizes (e.g. Cohen's d, Pearson's r), indicating how they were calculated

Our web collection on statistics for biologists contains articles on many of the points above.

Software and code

Policy information about availability of computer code

Data collection	Materials Studio 2018, NanoSight NTA v3.3, SparkControl v2.1, Leica Application Suite X 3.6.123246
Data analysis	GraphPad Prism v7.0c, Morpheus (Broad Institute, v0.1.1.1), Minitab v19.2020, ImageJ v1.53

For manuscripts utilizing custom algorithms or software that are central to the research but not yet described in published literature, software must be made available to editors and reviewers. We strongly encourage code deposition in a community repository (e.g. GitHub). See the Nature Research guidelines for submitting code & software for further information.

Data

Policy information about availability of data

All manuscripts must include a data availability statement. This statement should provide the following information, where applicable:

- Accession codes, unique identifiers, or web links for publicly available datasets
- A list of figures that have associated raw data
- A description of any restrictions on data availability

All data supporting the findings of this study are included within the article and its supplementary information, and are also available from the corresponding author upon reasonable request. Source data are provided with this paper.

Field-specific reporting

Life sciences study design

All studies must disclose on these points even when the disclosure is negative.

Sample size	A total of 12 colorectal cancer ascites samples (n = 12 individuals) were used in the study. Sample size depended on the availability of clinical samples.
Data exclusions	No data were excluded.
Replication	All measurements were performed at least three times and all data were presented. All attempts at replication were successful.
Randomization	Randomization was not applicable, as all samples were treated equally and not allocated into different experimental groups.
Blinding	All experiments were performed blinded from clinical survival data.

Reporting for specific materials, systems and methods

We require information from authors about some types of materials, experimental systems and methods used in many studies. Here, indicate whether each material, system or method listed is relevant to your study. If you are not sure if a list item applies to your research, read the appropriate section before selecting a response.

Materials & experimental systems

n/a	Involved in the study	
☐	☒	Antibodies
☐	☒	Eukaryotic cell lines
☒	☐	Palaeontology and archaeology
☒	☐	Animals and other organisms
☐	☒	Human research participants
☒	☐	Clinical data
☒	☐	Dual use research of concern

Methods

n/a	Involved in the study	
☒	☐	ChIP-seq
☒	☐	Flow cytometry
☒	☐	MRI-based neuroimaging

Antibodies

Antibodies used	CD24 antibody (eBioscienceTM, ThermoFisher Scientific, clone M1/69, catalog no. 14-0242-82)
Validation	Antibodies used have been validated by the manufacturer and each lot has been quality tested. Validation data are available on the manufacturers' website.<br>Anti-CD24 (eBioscience) has been tested by flow cytometric analysis of mouse splenocytes and thymocytes. (https://www.thermofisher.com/antibody/product/CD24-Antibody-clone-M1-69-Monoclonal/14-0242-82)

Eukaryotic cell lines

Policy information about cell lines

Cell line source(s)	Human glial cells (GLI36) and epithelial cells (A431) are from ATCC.
Authentication	All cell lines were authenticated by the manufacturer (ATCC): ATCC uses morphology, karyotyping, and PCR based approaches to confirm the identity of human cell lines and to rule out both intra- and interspecies contamination. These include an assay to detect species specific variants of the cytochrome C oxidase I gene (COI analysis) to rule out inter-species contamination, and short tandem repeat (STR) profiling to distinguish between individual human cell lines and rule out intraspecies contamination.
Mycoplasma contamination	All cell lines were tested negative for mycoplasma contamination.
Commonly misidentified lines<br>(See ICLAC register)	No commonly misidentified cell lines were used.

Human research participants

Policy information about studies involving human research participants

Population characteristics	Ascites samples were obtained from 12 colorectal cancer patients (7 male, 51–74 yo; and 5 female, 65-75 yo). Patients were deemed to have a good prognosis when the overall survival was more than ten months. Conversely, patients were determined to have a poor prognosis if the overall survival was less than five months.
Recruitment	No selection bias. Patients were recruited based on clinical diagnosis and survival profile as determined by independent gold-standard pathology and clinical evaluation. All subjects were recruited according to IRB-approved protocols after obtaining informed consent.
Ethics oversight	The study was approved by the National University Hospital (2016/01088), and SingHealth (2015/2479) Institutional Review Boards.

Note that full information on the approval of the study protocol must also be provided in the manuscript.
